# Loss of Fibronectin Fiber Tension in Glioblastoma is Associated with Microvascular Proliferations and Immune Cell Infiltration

**DOI:** 10.1002/advs.202416526

**Published:** 2025-09-19

**Authors:** Michele Crestani, Isabel Gerber, Arnaud Mieville, Katrin Frauenknecht, Theoni Maragkou, Tibor Hortobagyi, Viola Vogel

**Affiliations:** ^1^ Laboratory of Applied Mechanobiology Department of Health Sciences and Technology ETH Zurich Zurich 8006 Switzerland; ^2^ National Center of Pathology (NCP) Laboratoire National de Santé (LNS) Dudelange 3555 Luxembourg; ^3^ Institute of Tissue Medicine and Pathology University of Bern Bern 3008 Switzerland; ^4^ Department of Neuropathology University Hospital Zurich Zurich 8091 Switzerland

**Keywords:** brain tumor, interleukin IL‐7, mechanobiology, mechanosignaling, transglutaminase TG2

## Abstract

Glioblastoma, the most malignant primary glial brain tumor, is challenging to cure. Its progression involves profound changes in the tumor microenvironment that includes extensive remodeling of the extracellular matrix (ECM). However, the impact of ECM fiber tension on cell‐ECM crosstalk during brain tumor progression has not been explored. To address this, a thoroughly validated peptide tension probe (FnBPA5) is used to stain tissue cryosections and assess fibronectin fiber tension. It is found that in the infiltration zone, microvascular proliferations (MVPs) are filled with low‐tension fibronectin fibers alongside layered endothelial cells and alpha‐smooth muscle actin (α−SMA) expressing cells, in contrast to microvessels (MVs). Also brain tissue areas infiltrated by immune cells—specifically, CD45+/CD68+ macrophages—contained abundant untensed fibronectin fibers. Occasionally, leukocytes formed non‐encapsulated CD45+ aggregates that partially co‐localized with lymphatic‐endothelial‐like cells (LEC‐like). Significantly, the number of leukocytes is directly proportional to the density of collagen I/III fibers. These CD45+ aggregates have morphological similarities with Tertiary Lymphoid Structures (TLS). The discovery that fibronectin fibers lose their tension in MVPs and in immune cell‐infiltrated parenchyma is significant as ECM fiber tension is known to mechano‐tune molecular interactions. The findings introduce the potential for novel therapeutic strategies that exploit ECM mechanics to improve glioblastoma treatment outcomes.

## Graphical abstract

Researchers discovered that fibronectin fibers lose their tension in specific areas of glioblastoma brain tumors, accumulating in the lumen of proliferating blood vessels and in parenchyma next to infiltrating immune cell clusters. A novel molecular tension probe enables the mapping of fibronectin's fiber tension in glioblastoma for the first time. This suggests new mechano‐therapeutics for treating this aggressive brain cancer.

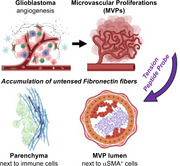

## 1 Introduction

Glioblastoma is the most prevalent malignant astrocytic primary brain tumor,^[^ ^1^ ^]^ with an approximate median survival of 14 months.^[^ ^1^, ^2^ ^]^ Standard treatments involve a combination of surgical resection, radiotherapy and concurrent chemotherapy,^[^ ^3^ ^]^ as no recent breakthroughs have yet surpassed these established protocols.^[^ ^4^, ^5^ ^]^ Integrins, which anchor cells to the extracellular matrix (ECM), initially emerged as promising therapeutic targets. This led to the clinical development of cyclic RGD pentapeptide to inhibit αvβ3 and αvβ5 integrins, namely Cilengitide; however, its failure in a phase III clinical trial highlighted gaps in our understanding of ECM‐driven tumor biology.^[^ ^6^ ^]^ This setback has reinforced the critical need to unravel the complex mechanobiological processes within the ECM that orchestrate glioblastoma's heterogeneous and highly invasive cellular landscape. Here, tumor cells interact intricately with non‐malignant cells and the ECM itself, sculpting a dynamic tumor microenvironment.^[^ ^7^, ^8^ ^]^ These interactions are instrumental in driving malignancy and are reflected in characteristic histopathological hallmarks that increasingly inform tumor grading—especially as digital histopathology refines diagnostic precision^[^ ^2^, ^9^, ^10^ ^]^ Well established in the literature, glioblastomas frequently exhibit blood vessel proliferation and attendant changes in perivascular niches, which encourage tumor plasticity, immune evasion, and metastatic spread.^[^ ^7^, ^8^, ^11^ ^]^ As these perivascular alterations provide valuable prognostic insight, with increasing precision due to the emerging field of digital histopathology,^[^ ^12^, ^13^ ^]^ the tumor microenvironment has become a focal point for AI‐assisted diagnostics and a promising target for innovative therapeutic strategies in glioblastoma.^[^ ^7^, ^8^, ^14^, ^15^ ^]^

**Figure 1:**
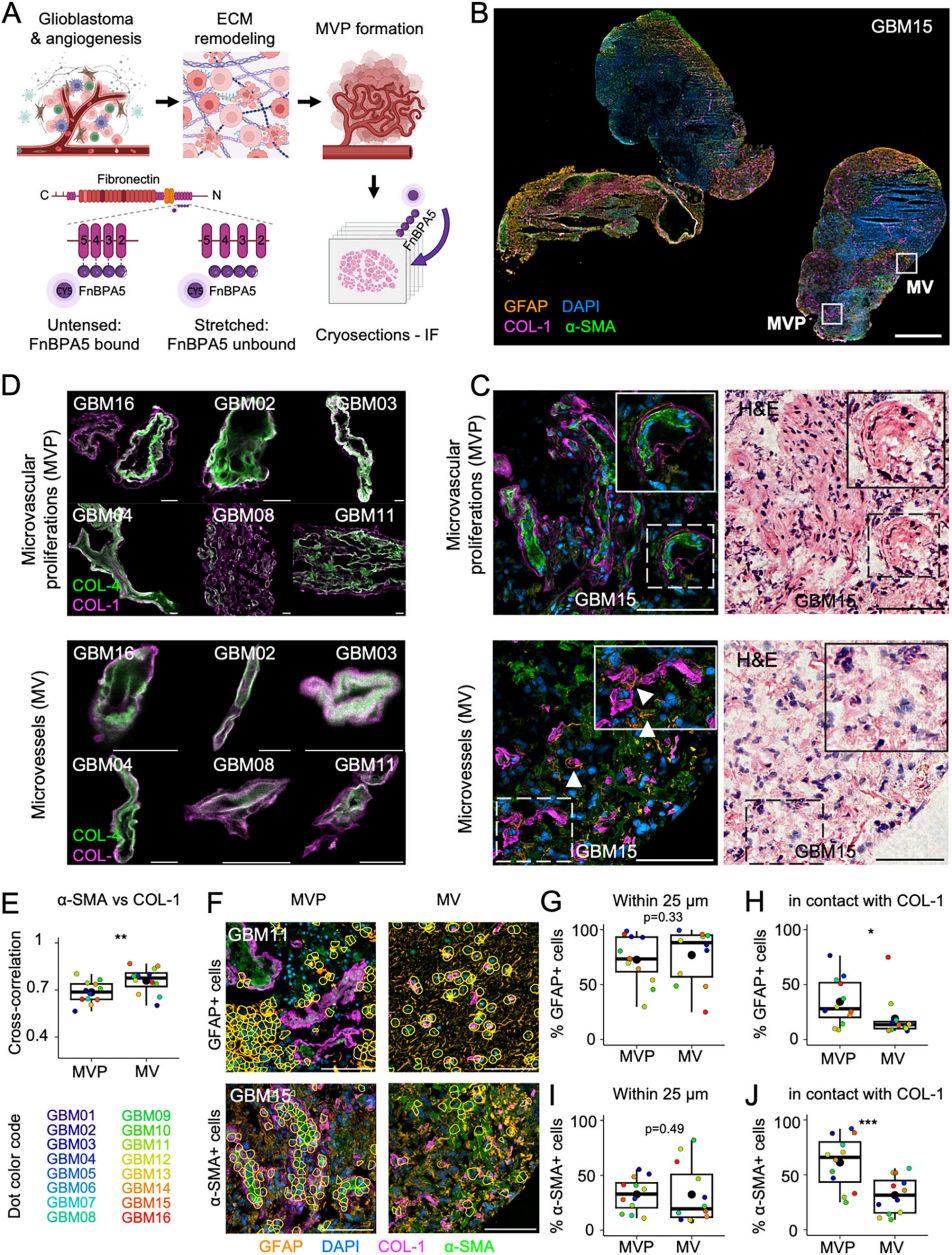
Microvascular proliferations (MVPs) are associated with GFAP‐ and α‐SMA‐expressing cells, which are in contact with a collagen IV basement membrane surrounded by a collagen I fiber rim. A) Schematic outlining the experimental procedure. B) Representative image from GBM15. Whole tissue immunostaining showing Glial Fibrillar Acidic Protein (GFAP, orange), DAPI (blue), collagen I (Magenta), alpha Smooth Muscle Actin (α‐SMA, green). Rectangles indicate the areas represented in C. Bar is 1mm. C) MVPs and MVs representative immunofluorescence images depicting GFAP, DAPI, collagen I and α‐SMA immunostaining (left) and H&E stains (right). Arrowheads in MV point to potential GFAP+ astrocytic end feet. Bars are 100 µm. D) Representative MVP and MV crops from single confocal slices of samples stained for collagen I (magenta) and collagen IV (green). Bars are 20 µm. E) Pixel‐by‐pixel cross‐correlation between α‐SMA and collagen I channels and the patient specific color code used throughout this work (pwr = 0.90). For this analysis, high‐resolution confocal images of MVs and MVPs were cropped at their borders. In each boxplot, dots are color coded as per legend after multiplexing over patients. In this figure, n = 26 MVPs (n = 1 for GBM2, GBM8, GBM9, GBM14; n = 3 for GBM3, GBM4, GBM10, GBM13, GBM15, GBM16; n = 4 for GBM11) and n = 38 MVs (n = 2 for GBM14, GBM16; n = 3 for GBM2, GBM3, GBM4, GBM10, GBM12; n = 4 for GBM13, GBM15; n = 5 for GBM9; n = 6 for GBM8) from 11 patients were used. F) α‐SMA+ and GFAP+ cells are highlighted with a yellow overlay, the collagen I margin with a magenta overlay. Bars are 100 µm. G,I) Percentage of GFAP+ G) and α‐SMA+ I) cells found within 25 µm of MVP/MV border, including the lumen. H, J) Percentage of GFAP+ (pwr = 0.48) H) and α‐SMA+ (pwr = 0.98) J) cells in contact (distance = 0 µm) or inside the lumen of MVPs and MVs. In all the boxplots, 1 dot represents the average from 1 patient sample and the bigger, black dots represent the mean value. In all the boxplots, one‐tailed unpaired t‐tests, non‐statistically significant p‐values are reported as 2‐digit numbers. Additional data can be found in Supplementary Figure S1 (Supporting Information).

A definitive diagnosis of glioblastoma relies on at least one of these molecular criteria: Telomerase Reverse Transcriptase (TERT) promoter mutation, Epidermal Growth Factor Receptor (EGFR) gene amplification and +7/−10 chromosome copy‐number alterations.^[^ ^16^, ^17^, ^18^ ^]^ Detecting these markers requires specialized assays, which are not always available in routine hospital settings. Consequently, pathologists often depend on visually discernible morphological features, which serve as the primary diagnostic hallmarks. These include necrosis, heightened proliferation rates, extensive tumor cell infiltration, nuclear atypia, palisading structures, and—central to our study—microvascular proliferations (MVPs).^[^ ^2^, ^19^, ^20^ ^]^ Histologically, MVPs are defined as “glomeruloid tufts of multilayered mitotically active endothelial cells together with smooth muscle cells/pericytes”,^[^ ^21^ ^]^ resulting in multi‐luminal vessels with irregular shapes (**Figure** **1**; Figure S2, Supporting Information).^[^ ^22^, ^23^, ^24^ ^]^ MVPs arise in response to hypoxic conditions,^[^ ^21^, ^25^ ^]^ which trigger upregulation of VEGF and thereby driving angiogenesis.^[^ ^26^ ^]^ While molecules such as angiopoietins, TGF‐α/β and TNF‐α are integral to in glioblastoma angiogenesis,^[^ ^27^ ^]^ studies in mouse models have demonstrated that VEGF alone is both sufficient for MVP induction and necessary for their maintenance.^[^ ^28^, ^29^ ^]^ In these murine experiments, adenoviral injection of VEGF164 led to increased vascular permeability, resulting in edema and extravascular fibrin deposition within 24 h. Numerous pre‐existing microvessels (MVs) then swelled into thin‐walled, pericyte‐deficient “mother vessels”. MVP precursors first appeared adjacent to VEGF164‐expressing cells by day 3, as rapidly dividing endothelial cells formed disorganized, fibroblast‐rich nodules that destabilized the mother vessels, transforming initially swollen single lumens into multiple irregular channels. In the later stages, pericytes were instrumental in assembling the basement membrane around endothelial cells, ultimately guiding the maturation process of MVPs.^[^ ^28^ ^]^

The ECM is an inherent and dynamic component of the tumor microenvironment, orchestrating cell fate through extensive, bidirectional crosstalk between cells and the ECM itself—a process modulated by integrins and other ECM‐binding proteins.^[^ ^7^, ^8^, ^11^, ^14^ ^]^ While excessive collagen fiber deposits can be visualized label‐free using Second Harmonic Generation (SHG) microscopy in biopsy samples,^[^ ^30^ ^]^ other ECM proteins—primarily secreted by endothelial cells and pericytes—include collagen IV (COL4A1), elastin, laminin, and fibronectin. In glioblastoma, these proteins are predominantly localized along the basement membranes of blood and lymphatic vessels.^[^ ^31^, ^32^, ^33^, ^34^ ^]^ The brain parenchymal ECM, in contrast, is rich in glycosaminoglycans (GAGs) such as hyaluronan (HA) and chondroitin sulfates (CS), as well as glycoproteins,^[^ ^35^ ^]^ but is largely devoid of fibrillar proteins. Astrocytes—the most abundant glial cells—are chiefly responsible for creating and remodeling this parenchymal ECM.^[^ ^36^ ^]^ Under physiological conditions, astrocytes maintain the integrity of the blood‐brain barrier and regulate metabolite homeostasis.^[^ ^37^, ^38^ ^]^ However, in central nervous system (CNS) pathologies like glioblastoma, astrocytes undergo molecular, cellular, and functional changes—becoming reactive astrocytes—in a process known as astrogliosis. This transformation is marked by overexpression of intermediate filament proteins such as Glial Fibrillary Acidic Protein (GFAP), as well as increased production of various ECM components, including collagens. Important in this context, neoplastic glial cells with astrocytic morphology also express GFAP.^[^ ^39^ ^]^ Within the tumor microenvironment, reactive astrocytes activate JAK/STAT signaling—often via interleukin‐6 secretion—triggering the expression of vascular endothelial growth factor (VEGF) and thereby promoting angiogenesis and the formation of MVPs. Interleukin‐6 also upregulates matrix metalloproteinases (MMPs), which drive ECM degradation.^[^ ^36^ ^]^ Vascular and ECM remodeling is not limited to astrocytes; endothelial cells, pericytes, and smooth muscle cells all contribute to this process.^[^ ^2^, ^19^, ^40^, ^41^ ^]^ Although Cancer Associated Fibroblasts (CAFs) were long considered absent from the brain, recent studies have identified alpha smooth muscle actin (α‐SMA) and collagen I (COL1A1) expressing CAFs, where they play pivotal roles in remodeling the perivascular tumor microenvironment.^[^ ^42^, ^43^ ^]^ In the tissue bulk, or parenchyma, elevated levels of HA, GAGs, SPARC, and tenascin‐C (TNC) facilitate tumor cell invasion,^[^ ^33^, ^35^, ^44^, ^45^, ^46^, ^47^ ^]^ foster the development of immune‐suppressive environments,^[^ ^48^, ^49^ ^]^ and promote angiogenesis.^[^ ^50^ ^]^ Despite the brain being among the softest of all organs, ECM stiffness increases with tumor grade, driven by ongoing ECM remodeling. This stiffening is detectable in vivo using Shear Wave Elastography (SWE) in brain tumor patients,^[^ ^51^ ^]^ or ex vivo through Atomic Force Microscopy (AFM) indentation of tissue slices.^[^ ^52^ ^]^ While significant advances have been made in correlating physical and molecular changes with glioblastoma progression,^[^ ^7^, ^8^, ^51^, ^52^, ^53^ ^]^ it remains unknown how these pro‐oncogenic ECM modifications influence the tensile state of individual ECM fibers, and how this, in turn, correlates with the infiltration capacity of various immune cell populations.

ECM remodeling creates a pro‐oncogenic environment that governs both the infiltration and phenotypic polarization of immune cells.^[^ ^54^, ^55^, ^56^ ^]^ In solid tumors such as glioblastoma, the ECM forms a dense and formidable barrier, physically impeding immune cell infiltration.^[^ ^14^, ^55^, ^57^, ^58^ ^]^ Low‐grade gliomas are predominantly infiltrated by “M1”‐like pro‐inflammatory microglia (the resident immune cells of the CNS), whereas in glioblastoma, macrophages are reprogrammed toward “M2”‐like pro‐tumorigenic phenotypes as the tumor architecture disrupts local vasculature.^[^ ^59^ ^]^ Furthermore, increased leukocyte infiltration correlates with the build‐up of Tertiary Lymphoid Structures (TLS) in human glioblastoma.^[^ ^60^, ^61^ ^]^ TLS are organized, non‐encapsulated aggregates of immune cells that develop postnatally within inflamed, non‐lymphoid tissues.^[^ ^62^ ^]^ Although lymphatic involvement in glioblastoma has historically received little attention, recent studies have brought it to the forefront. Latest evidence showed that VEGF‐C ectopic expression in glioblastoma murine models has potentiated the lymphatic‐system mediated immunosurveillance.^[^ ^63^ ^]^ In the human brain parenchyma, lymphatic endothelial‐like cells (LEC‐like), marked by high expression of Lymphatic Vessel Endothelial Hyaluronan Receptor 1 (LYVE1), promote glioblastoma stemness through cytokine‐driven cholesterol metabolism.^[^ ^64^ ^]^ Despite these advances, it remains unclear how ECM remodeling and the mechanical properties of individual ECM fibers may direct, or be influenced by, the pathophysiology of lymphatic/LEC‐like cells and the formation of TLS in glioblastoma.

Among the fibrillar proteins significantly overexpressed in glioblastoma, fibronectin contains a plethora of binding sites for ECM proteins, growth factors and cells.^[^ ^65^ ^]^ Until recently, nothing was known regarding the tension of individual ECM fibers due to the absence of suitable nanoscale measurement tools. However, emerging research now indicates that modulations in fibronectin fiber tension are pivotal to the development of a pro‐oncogenic extracellular matrix.^[^ ^56^, ^66^, ^67^, ^68^ ^]^ This is particularly significant, as fibronectin fiber tension not only alters th Young's modulus upon fiber stretching,^[^ ^69^ ^]^ but also modulates molecular interactions, as fiber stretching can mechano‐regulate binding site exposure^[^ ^65^, ^70^ ^]^ and might switch the affinity of molecular binding partners. This mechano‐chemical switching disrupts multivalent binding motifs, or reveals hidden ones, thereby tuning the binding of growth factors, cytokines and other ECM‐binding proteins,^[^ ^65^, ^69^, ^71^, ^72^, ^73^, ^74^ ^]^ including Transglutaminase type 2 (TG2)^[^ ^75^ ^]^ and Interleukin‐7 (IL‐7).^[^ ^76^ ^]^ Stretching fibronectin fibers also extends the distance between the synergy site and RGD loop, situated on the type III modules nine and ten, respectively, thereby reducing the affinity of α5β1 integrins to fibronectin.^[^ ^71^, ^73^, ^77^ ^]^ Thus, the mechano‐chemical regulation of ECM fiber strain might mechanically tune the cell‐ECM crosstalk.^[^ ^65^, ^70^ ^]^ How these processes regulate glioblastoma progression remains elusive. TG2, for example, modulates the tumor microenvironment, influencing tumor initiation, growth, and metastasis.^[^ ^78^, ^79^, ^80^ ^]^ In vitro single fiber stretch assays have demonstrated that TG2 in the closed conformation binds with higher affinity to untensed than to highly tensed fibronectin fibers.^[^ ^75^ ^]^ Thus, the mechano‐regulated exposure or activation of binding sites is highly relevant for how fibronectin fiber tension shapes the biochemical composition of the tumor microenvironment.^[^ ^65^, ^70^ ^]^ Importantly, increased tension in fibronectin fibers can disrupt multivalent binding motifs—such as the stretch‐induced destruction of bacterial adhesin epitopes within the N‐terminal fibronectin type I modules (FnI_2‐5_). To exploit this, we leveraged adhesion peptides, evolved by bacteria to recognize cleaved fibronectin fibers for host invasion at wound sites and developed the peptide FnBPA5 to read out fibronectin fiber tension in tissue cryosections and in vivo.^[^ ^68^ ^]^ The peptide probe was carefully validated: steered molecular dynamic simulations showed atomistic structural details how the binding epitope on FnI_2‐5_ gets destroyed upon stretching,^[^ ^72^, ^81^ ^]^ while in vitro fibronectin fiber stretch assays revealed that FnBPA5's affinity for fibronectin diminishes rapidly upon fiber stretching.^[^ ^72^, ^81^ ^]^ Finally, in cell culture experiments, using FnBPA5 to read out fibronectin's fiber strain cross‐correlated well with our previously developed fibronectin‐FRET‐probe.^[^ ^68^, ^82^, ^83^ ^]^ FnBPA5 binds to untensed fibronectin fibers with nM affinity in murine breast, adenocarcinoma and infected lymph nodes tissue cryosections but far less to fibronectin fibers in healthy organs which indicates that they are stretched in healthy ECM.^[^ ^56^, ^65^, ^67^ ^]^

As nothing was known regarding fibronectin's fiber tension in glioblastoma and whether it plays a functional role, it is essential to correlate loci with low fibronectin fiber tension with other key molecular and morphological hallmarks of the disease. In this study, we investigated whether glioblastoma is characterized by the presence of low‐tension fibronectin fibers—a hypothesis our findings confirmed. Untensed fibronectin fibers were identified within MVPs of human glioblastoma tissue cryosections (Figure 1A), whereas the surrounding soft brain parenchyma remained largely fibronectin‐free.^[^ ^35^ ^]^ Building on our recent discovery that low‐tension fibronectin fiber tracks in murine breast tumors are densely populated with M2 macrophages,^[^ ^56^ ^]^ we further explored whether reduced fibronectin fiber tension correlates with the spatial distribution of infiltrating immune cells (Figure 1A). Clinically, structures such as tertiary lymphoid structures (TLS)^[^ ^60^, ^61^ ^]^ and lymphatics^[^ ^63^ ^]^ have generated increased attention as targets in adjuvant immunotherapies. A distinctive aspect of our work is the identification of loci where TLS and lymphatic structures colocalize with untensed fibronectin fibers. Collectively, our results suggest that the loss of fibronectin fiber tension in glioblastoma is intrinsically linked to the presence of MVPs and the infiltration of immune cells.

## 2 Results

To study ECM remodeling processes that are associated with the formation of MVPs, immune cell infiltration, TLS and LEC‐like cells, human tumor tissue cryosections of 16 patients diagnosed with glioblastoma were used, all IDH‐wildtype, CNS World Health Organization (WHO) grade 4 and above the age of 50. The salient clinical features of these glioblastoma samples are summarized in **Table** **1**. Histological immunostainings were compared with hematoxylin and eosin (H&E) stains (Figure 1C; see Experimental Section). A histopathological overview of H&E stains shows typical pathological transformations, including diffuse, necrotic, MVPs, MVs and tumor cell pseudopalisades (Figure S2, Supporting Information). To provide patient‐specific information, the boxplot dots of all multiplexed analyses are color coded (see Table 1 and Figure 1E), whereby each dot represents an average over the parameter sampled several times within the tissue slice. Images were analyzed with the help of neuropathologists for the identification of MVPs, which were always differentiated and compared with healthy MVs within the same tumor.

**Table 1:** Overview of brain tumor tissue samples and relevant clinical data. All samples were clinically diagnosed as Glioblastoma, Isocitrate dehydrogenase (IDH) wild type (CNS grade 4, integrative diagnosis WHO 2021). The O6‐Methylguanine‐DNA Methyltransferase (MGMT) promoter methylation correlates with better patient survival.

Tumor ID	Age	Sex	Location	Histopathological classification	MGMT Promoter methylation
GBM01	73	M	Occipital	Glioblastoma, IDH wild type	Positive
GBM02	57	M	Right frontal lobe	Glioblastoma, IDH wild type	Negative
GBM03	76	M	Right parietal lobe	Diffuse astrocytic glioma with signs of anaplasia	Positive
GBM04	76	F	Left parietal lobe	Glioblastoma, IDH wild type	Positive
GBM05	72	M	Right parietal lobe	Glioblastoma, IDH wild type	Negative
GBM06	70	M	Temporal lobe	Glioblastoma, IDH wild type	Negative
GBM07	72	M	Occipital	Glioblastoma, IDH wild type	Positive
GBM08	53	F	Right temporal lobe	Glioblastoma, IDH wild type	N.A.
GBM09	75	F	N.A.	Glioblastoma, IDH wild type	Positive
GBM10	83	F	N.A.	Glioblastoma, IDH wild type	Negative
GBM11	61	M	Left parietal lobe	Glioblastoma, IDH wild type	Negative
GBM12	56	M	Corpus callosum bilateral	Glioblastoma, IDH wild type	Negative
GBM13	58	F	Right hemisphere	MGlioblastoma, IDH wild type	Negative
GBM14	78	M	Right parietal lobe	MGlioblastoma, IDH wild type	Positive
GBM15	59	F	Right temporal lobe	Diffuse astrocytic glioma with signs of anaplasia	Negative
GBM16	85	F	Right medial temporal lobe	Diffuse astrocytic glioma with signs of anaplasia	Negative

### 2.1 Microvascular proliferations (MVPs) are Associated with GFAP‐ and α‐SMA‐Expressing Cells, which are in Contact with a Collagen IV Basement Membrane Surrounded by a Collagen I Fiber Rim

Given the role of GFAP‐expressing cells in promoting ECM degradation and angiogenesis, glioblastoma tissues were immunostained with a panGFAP antibody. This antibody cannot distinguish between reactive astrocytes and neoplastic glial cells of astrocytic morphology, so we henceforth refer to them as GFAP+ cells. α‐SMA and COL1A1 antibodies were concomitantly used, as α‐SMA is expressed by smooth muscle cells, pericytes and CAFs.^[^ ^2^, ^41^, ^42^ ^]^ In all tissue stains, GFAP and α‐SMA were detected in proximity to collagen I (Figure 1B). Areas low in or with a diffused DAPI signal, indicative of edema or necrosis, were low in GFAP and α‐SMA. These areas are marked by asterisks in the H&E image (Figure S1A, Supporting Information). Reactive astrocytes and α‐SMA‐expressing cells overexpress ECM molecules such as collagen I/IV.^[^ ^36^, ^43^, ^84^ ^]^ Co‐immunostaining of whole glioblastoma tissues with COL4A1 and COL1A1 revealed that the main basement membrane marker collagen IV^[^ ^31^, ^32^, ^33^, ^34^ ^]^ is surrounded by a collagen I rim in MVPs and MVs (Figure 1D). This suggests that collagen I expressing cells surround the exterior of the vessels and that COL1A1 can be used to detect the outermost vessel border. SHG revealed an overlap with immunostained COL1A1, hence confirming these results (Figure S1C, Supporting Information).

As expected, zoom‐ins of MVPs and MVs showed that GFAP+ cells were localized mainly outside of MV/MVP basement membranes, as revealed by GFAP and COL1A1 stainings, respectively (Figure 1C). The mean GFAP signal stayed constant as distance from MV border increased, while significantly grew as distance from MVP border increased (Figure S1D,E, Supporting Information). Since astrocytes build up a homogeneously distributed network in the brain parenchyma that ensures blood‐brain barrier integrity,^[^ ^37^, ^85^ ^]^ a constant GFAP signal around MV might suggest the presence of such a network around MVs, as expected, potentially forming direct contacts via end‐feet (arrowheads in Figure 1C), unlike for MVPs. However, an equally high fraction of GFAP+ cells was detected in a 25 µm wide rim surrounding either MV or MVP borders, with 73 ± 22% and 77 ± 25%, respectively (Figure 1G), as identified from DAPI stains (Figure 1F). Surprisingly though, the percentage of GFAP+ cells in contact with basement membranes was higher in MVPs than in MVs (34 ± 21% vs 19 ± 19%; Figure 1H), indicating a higher presence of reactive astrocytes or neoplastic glial cells in proximity to MVPs than MVs (Figure S1I, Supporting Information). As reactive astrocytes are involved in angiogenesis and ECM degradation,^[^ ^36^ ^]^ their high presence in contact with MVPs suggests that they could play an active role in MVP formation.

In contrast, α‐SMA was localized mostly inside the MVPs (Figure 1C), consistent with previous reports,^[^ ^29^, ^41^ ^]^ whereas no preferential localization in proximity to MV was seen (Figure 1C). This is further corroborated by the constant α‐SMA signal as the distance from the MV and MVP borders increased (Figure S1F, Supporting Information). As for GFAP, the α‐SMA signal associated with single cells showed no differences in the percentage of α‐SMA+ cells within 25 µm from MVPs and MVs, with 33 ± 15% and 33 ± 26% (mean ± SD), respectively (Figure 1I). Yet, the majority of α‐SMA+ cells resided inside, or externally in direct contact with MVPs (61 ± 24%), while it reached only 31 ± 17% for MVs (Figure 1J). This suggests a higher presence of smooth muscle cells, pericytes and CAFs in proximity to MVPs than MVs (Figure S1H, Supporting Information).

Normalized cross‐correlation analysis between α‐SMA and COL1A1 pixels of MVP and MV crops revealed a significantly lower co‐localization between α‐SMA and COL1A1 in MVPs than in MVs (Figure 1E), whereby the corresponding mean COL1A1 intensities were significantly lower in MVPs (Figure S1G, Supporting Information). Data were multiplexed over 12 samples (n = 26 MVPs, n = 38 MVs), with Figure S1B (Supporting Information) showing representative cropped MVs and MVPs. Taken together, these results show that α‐SMA expressing cells were preferentially localized inside MVPs, while collagen IV rich basement membranes were surrounded by GFAP‐expressing cells and a collagen I rim, typically produced by smooth muscle cells, pericytes, CAFs and reactive astrocytes.

### 2.2 The Vessel Walls of MVs were Rich in Collagen IV and Stretched Fibronectin Fibers, while Untensed Fibronectin Fibers were Solely Associated with MVPs

**Figure 2:**
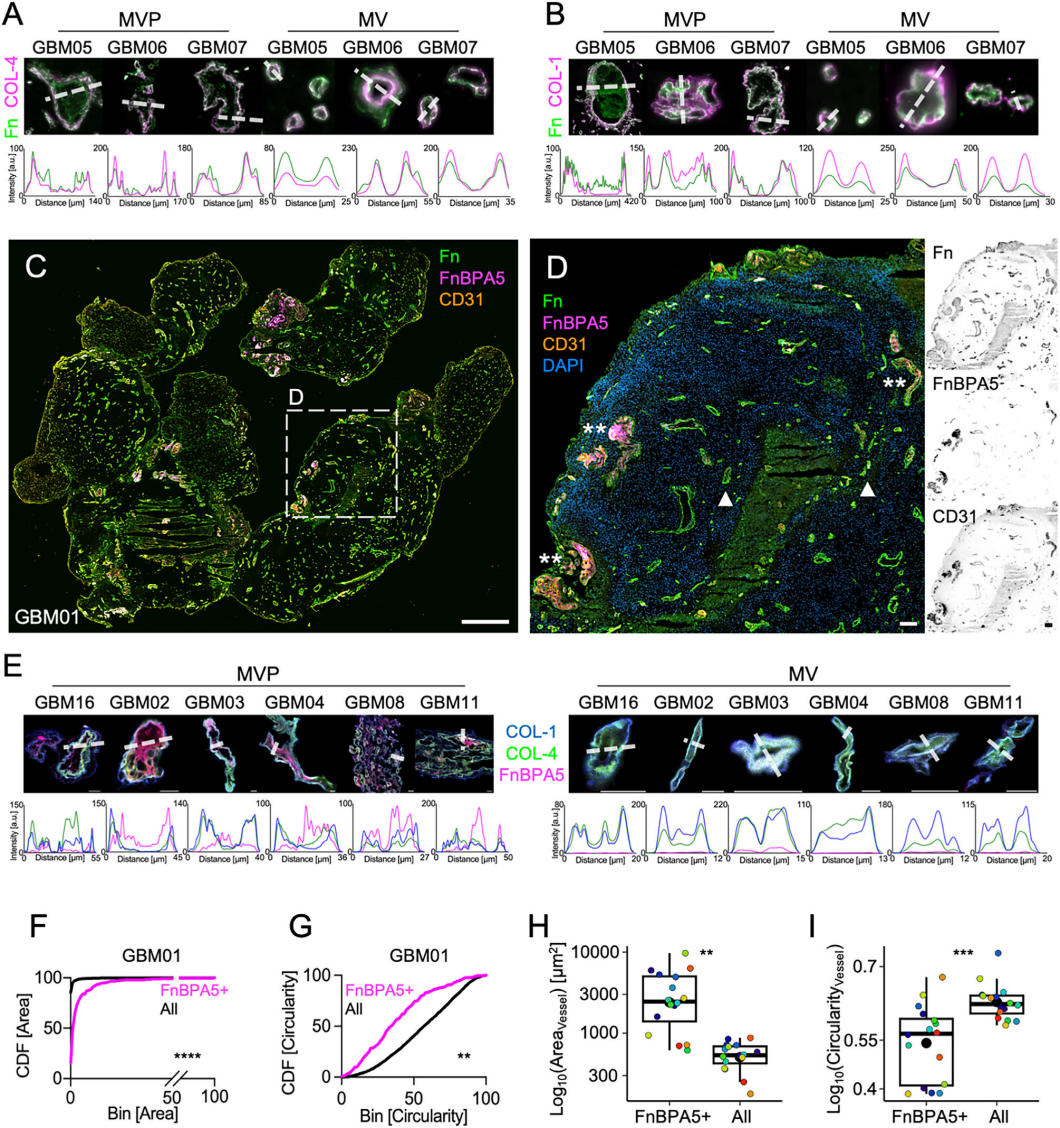
The vessel walls of MVPs and MVs are rich in collagen IV and stretched fibronectin fibers, while untensed fibronectin fibers are solely associated with interior of MVPs. A) Representative MVP and MV crops from whole tissue scans of samples stained for collagen IV (magenta) to localize the basement membrane and a polyclonal fibronectin antibody (green) obtained from 3 selected glioblastomas complemented by line profile plots. Bars are 20 µm. B) Representative MVP and MV crops from whole tissue scans of samples stained for collagen I (magenta) and fibronectin (green) obtained from 3 selected glioblastomas complemented by line profile plots. Bars are 20 µm. C) Representative image from GBM01 whole tissue scan showing immunostainings for fibronectin (Fn, green), CD31 (orange), and untensed fibronectin fibers as stained by the fibronectin fiber tension probe FnBPA5 (magenta). Rectangles indicate the areas represented in D, E. Bar is 1mm. D) Representative images indicating vessel structures enriched in untensed fibronectin fibers. Asterisks indicate MVPs, while arrowheads point to MVs. Bar is 200 µm. Single channels are displayed on the right (black and white). E) Representative MVP (left) and MV (right) crops from single confocal slices of samples stained for collagen I (blue), collagen IV (green), FnBPA5 (magenta) plus the respective line profile plots along the dotted lines. F,G) Cumulative Distribution Functions (CDF) of vessel area H) and circularity I) in GBM01. Kolmogorov‐Smirnov tests. H,I) Mean area (pwr = 1) H) and circularity (pwr = 1) I) of FnBPA5+ versus all vessels. n = 16 patients. One‐tailed unpaired t‐tests. In all the boxplots, 1 dot represents the average from 1 patient sample and the bigger, black dots represent the mean value. Additional data can be found in Figures S2–S5 (Supporting Information).

As fibrillar proteins like laminins, collagen IV and fibronectin are found almost entirely along the basement membrane of vessels,^[^ ^31^, ^32^, ^33^ ^]^ we initially co‐stained glioblastoma samples with a fibronectin polyclonal antibody, which identifies all fibronectin isoforms, irrespective of their tension, and COL4A1 to localize fibronectin with respect to collagen IV. Line scan analysis showed that fibronectin decorated the surroundings of collagen IV in the basement membrane with thin fibers in MVPs and MVs and occasionally filled the lumen of MVPs (**Figure** **2**A; Figure S3A, Supporting Information). Conversely, COL1A1 stainings and line scan analyses confirmed that collagen I typically formed a shaft surrounding the Fibronectin‐ and collagen IV‐rich basement membrane in MVs (Figure 2B; Figure S3B, Supporting Information), which is expected as pericytes and smooth muscle cells surround vessels and assemble collagen I. When single MVPs had multi‐lumina, COL1A1 filled the space in between them (Figure 2B, second column).

To ask whether the presence of MVPs goes along with changes in fibronectin fiber tension, we co‐stained 16 glioblastoma tumor tissue sections with our FnBPA5 tension probe (Figure S4, Supporting Information) to localize untensed fibronectin fibers,^[^ ^67^, ^68^ ^]^ while the fibronectin polyclonal antibody binds independently of fibronectin's tensional state. The vasculature structures were identified by co‐staining with CD31, a cell adhesion marker expressed by endothelial cells^[^ ^86^ ^]^ (Figure 2C; Figure S3C, Supporting Information). High CD31 signal intensities were detected inside the vessel walls of large glomerular MVPs (Figure 2D; Figure S2D, Supporting Information, asterisks). Fibronectin was found in all vessel walls and necrotic areas. FnBPA5 stained highly necrotic regions and glomerular MVPs, where it was particularly evident within the vessel walls of the largest MVPs (Figure 2D; Figure S2D, Supporting Information, asterisks). FnBPA5 did not bind to MVs (Figure 2D, arrowheads). This suggests that big MVPs were frequently enriched in untensed Fibronectin, whereas the smaller and more circular MVs contain mostly stretched fibronectin fibers. Unlike in MVs (Figure 2E, right), line scans across MVPs confirmed that untensed fibronectin fibers were enveloped by collagen IV rich basement membranes (Figure 2E, left), with stretched fibronectin fibers located only immediately adjacent to the collagen IV borders.

To quantitatively verify the association between untensed fibronectin fibers and MVPs, threshold‐based classification of FnBPA5+ vessels in the GBM01 tissue slice (a thresholding example from an inset in GBM01 is in Figure S2E, Supporting Information) revealed that structures positive for FnBPA5 were bigger in size and less circular, as represented with cumulative distribution functions (CDF; Figure 2F,G). As large areas and glomerular, non‐circular shapes are typical of MVPs, this strikingly was a common signature of all 16 glioblastoma samples (Figure 2H,I) and confirmed with FnBPA5 mean intensity sampling, which was high in MVPs and nearly zero in MVs (Figure S3F, Supporting Information). We found no correlation between MGMT methylation status (MGMT promoter methylation correlates with better patient survival) or patient age and MVP area or MVP/MV FnBPA5 intensity (Figure S3G–J, Supporting Information). In contrast, no untensed fibronectin fibers were found in vessels in the Cerebellum and Dura Mater from healthy patients, even in high‐resolution confocal images (Figure S5, Supporting Information), nor a randomly scrambled negative control peptide (scrFnBPA5‐Cy5) showed any binding to the healthy brain tissue (Figure S3F, Supporting Information). Taken together, our data show that untensed fibronectin fibers are predominantly associated with MVPs in glioblastoma.

### 2.3 MVP Basement Membranes Enclosed Layered Endothelial Cells and Untensed Fibronectin Fibers

**Figure 3:**
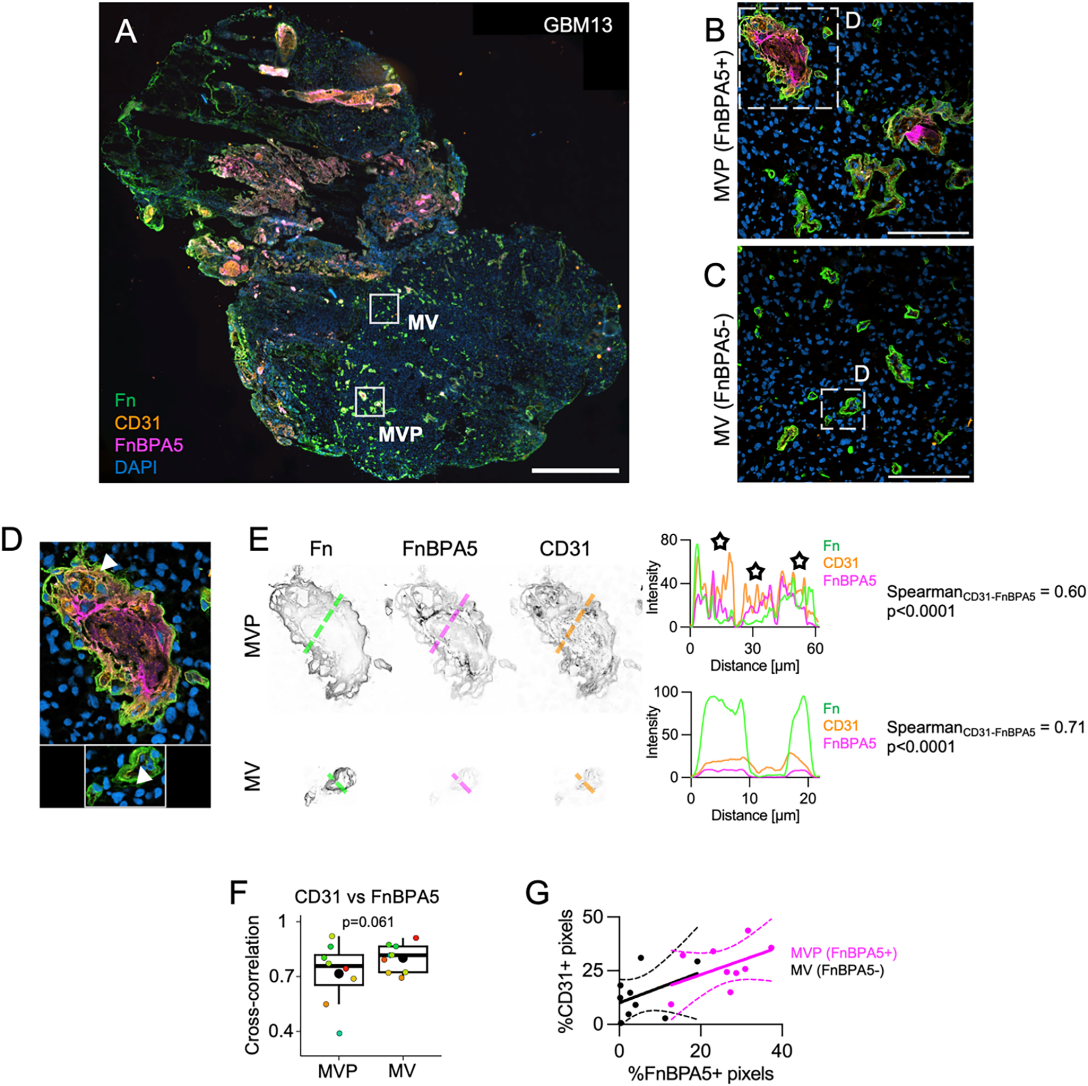
MVP basement membranes enclose layered endothelial cells and untensed fibronectin fibers. A) Representative image from GBM13 whole tissue scan showing immunostaining for DAPI (blue), polyclonal fibronectin antibody (Fn, green), CD31 (orange), and untensed fibronectin fibers as stained by the tension probe FnBPA5 (Magenta). Rectangles indicate the areas represented in B, C. Bar is 1mm. B, C) Representative areas of MVs and MVPs, poor (FnBPA5‐) and rich in untensed fibronectin fibers (FnBPA5+)(C), in B9 and C) respectively. Bars are 100 µm. D) Representative crops of single MVs and MVPs. Arrowheads point to a CD31+ multilayered endothelial area in a MVP and to a single, thin endothelial layer in a MV. E) Single channel images of fibronectin, CD31 and FnBPA5 immunostainings and line profile plots from the MVPs and MVs in G). F) Pixel‐by‐pixel cross‐correlation between CD31 and FnBPA5 channels (pwr = 0.75). One‐tailed unpaired *t*‐test. 1 dot represents the average from 1 patient sample, and the bigger, black dots represent the mean value. G) Percentage of CD31+ pixels plotted against the percentage of FnBPA5+ pixels. Pixels were sampled within MVPs rich in FnBPA5+ (purple) and MVs poor in FnBPA5‐ (black). Lines represent linear regression ± confidence intervals. Additional data can be found in Figure S6 (Supporting Information).

Since MVPs contain α‐SMA‐expressing cells in their lumen (Figure 1) together with a large fraction of fibronectin fibers that had lost their tension (Figure 2), we next investigated the spatial relationship between CD31+ endothelial cells and untensed fibronectin fibers (**Figure** **3**A). Zoom‐ins of MVPs show a stratified CD31‐rich endothelium with high nuclear density, the hallmark of MVPs,^[^ ^21^, ^22^, ^23^ ^]^ surrounded by a green rim of stretched fibronectin fibers (Figure 3B; arrowhead in Figure 3D top). In contrast, MVs had a lower density of nuclei and were mostly stained only for fibronectin (Figure 3C) and a thin CD31+ layer (arrowhead in Figure 3D bottom). As MVPs result from pathological angiogenesis, where endothelial cells form multi‐layered disorganized walls, a higher CD31 intensity in FnBPA5+ MVPs (Figure S6A, Supporting Information) supports the high mitotic activity of MVP endothelial cells.^[^ ^2^, ^19^, ^40^, ^86^ ^]^ This suggests that the high mitotic activity of MVP endothelial cells, which is a manifestation of hyperplasia, correlates with the appearance of untensed fibronectin fibers.

Moreover, line scans showed that FnBPA5 intensity peaks (stars in the graph of Figure 3E) were localized inside the MVP lumen and juxtaposed to CD31 intensity peaks, while not present inside MVs (Figure 3E). Juxtaposition is also seen between endothelial cells and untensed fibronectin fibers (Figure 3E; Figure S6B, Supporting Information, note the lower Spearman correlation coefficient in MVPs), which was particularly evident in 5/8 more patients (stars in the graphs of Figure S6C, Supporting Information) and never detected in MVs. A lower FnBPA5 versus CD31 co‐localization in MVPs than in MVs corroborated these findings throughout the 9 patients analyzed (Figure 3F). Strikingly, CD31 and FnBPA5 pixels correlated linearly in MV and MVP image crops (Figure 3G, 1 point per sample). Together, these data suggest that fibronectin fibers are tensed near basement membranes of MVs, while they had lost their tension in proximity to stratified endothelia in the lumen of pathologically transformed MVPs.

### 2.4 Macrophages Mostly Infiltrated the Parenchyma in Locations where Fibronectin Fibers are Structurally Disorganized and have Lost their Tension

**Figure 4:**
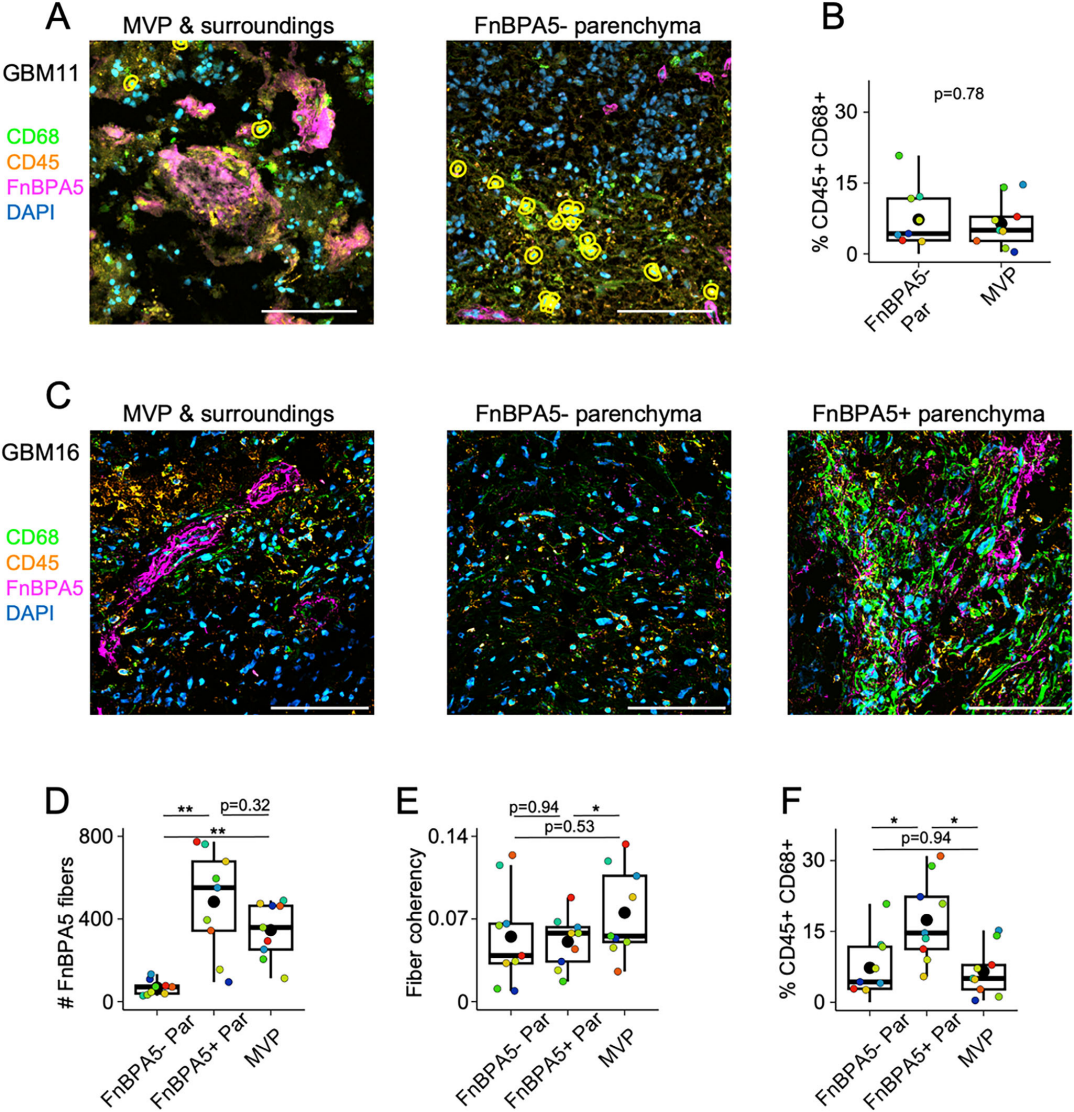
Macrophages occasionally infiltrate the parenchyma in locations where fibronectin fibers are structurally disorganized and have lost their tension. A) CD45+/CD68+ macrophages are highlighted with a yellow overlay in MVPs found in parenchyma poor of untensed fibronectin fibers (FnBPA5‐). B) Percentage of CD45+/CD68+ macrophages found in the proximity of MVPs and in parenchyma poor of untensed fibronectin fibers (FnBPA5‐) from 9 patient samples. Two‐tailed unpaired t‐test, non‐statistically significant p‐value is reported. C) Representative confocal images for tumor GBM16 stained for CD45 (orange), CD68 (green), FnBPA5 (magenta) and DAPI (blue). Fibronectin fibers are organized in a vessel‐like fashion (MVP, left). In other locations, the parenchymal ECM is poor in untensed fibronectin fibers (middle), or rich in disorganized, untensed fibronectin fibers (right). D,E) Untensed fibronectin fiber analysis for number of fibers (pwr_FnBPA5‐ FnBPA5+_ = 1; pwr_FnBPA5‐ MVP_ = 1). D) and coherency, which indicates the alignment of fibers with respect to the image dominant direction of orientation (0 to 1; pwr_FnBPA5+ MVP_ = 0.60) E). RM one‐way ANOVA test with Geisser‐Greenhouse correction and Turkey's multiple comparison test. F) Percentage of CD45+/ CD68+ macrophages populating parenchyma poor of untensed fibronectin fibers (FnBPA5‐) is small compared to FnBPA5‐rich parenchyma, MVPs (pwr_FnBPA5‐ FnBPA5+_ = 0.87; pwr_FnBPA5+ MVP_ = 0.69). RM one‐way ANOVA test with Geisser‐Greenhouse correction and Turkey's multiple comparison test. Bars in all panels are 100 µm. In all the boxplots, 1 dot represents the average from 1 patient sample and the bigger, black dots represent the mean value. Additional data can be found in Figure S7 (Supporting Information).

In glioblastoma, ECM remodeling is known to regulate immune cell functions and is associated with endothelial vessel wall breakdown and the formation of perivascular niches that harbors “M2”‐like protumorigenic macrophages.^[^ ^8^, ^55^, ^87^ ^]^ We showed previously in a murine breast cancer model that tumor progression corrupted blood vessels (via an Endothelial‐to‐Mesenchymal Transition (EndoMT), a process of transdifferentiation where endothelial cells gradually adopt the phenotypic characteristics of mesenchymal cells.^[^ ^54^ ^]^ In the resulting matrix tracks, low tension fibronectin fibers were found in the inner lumen together with collagen fiber bundles and enriched in M2 macrophages.^[^ ^54^ ^]^ To ask whether similar processes might be involved in association with MVPs in human glioblastoma, we immunostained our samples with CD45, CD68 and FnBPA5. CD45 is a pan‐leukocyte marker, excluding erythrocytes and platelets.^[^ ^88^ ^]^ CD68 is expressed by monocytes, neutrophils, basophils, microglia, and, importantly, is the most‐widely used marker for all macrophages.^[^ ^89^ ^]^ Macrophages, however, did not preferentially co‐localize with low‐tension fibronectin fibers inside of MVPs, as revealed using high resolution confocal images co‐stained for CD45 and CD68 (**Figure** **4**A). Quantification of the percentage of CD45+/CD68+ macrophages from 9 patients was surprisingly not different between MVPs plus their surroundings as compared to the FnBPA5‐poor parenchyma (Figure 4A,B).

Nevertheless, we observed one exception in GBM16, originating from the oldest patient of our dataset, which harbored hotspots of intense CD68 stain in FnBPA5‐rich parenchyma. Here, remarkably, untensed fibronectin fibers were sparse and highly disorganized, devoid of recognizable vessel morphologies (Figure 4C, right). Conversely, FnBPA5 signal was organized in vessel‐like fashion in MVP and really dim in FnBPA5‐poor parenchyma, as expected (Figure 4C middle, left, respectively; Figure S7A,B, Supporting Information).

Quantification of number of untensed fibronectin fibers confirmed that FnBPA5‐rich parenchyma contained the highest number of untensed fibronectin fibers in 9 glioblastomas (Figure 4D). Vice versa, untensed fibronectin fibers in MVPs exhibited a greater coherency, which indicates preferential orientation along a dominant direction, than in FnBPA5‐rich and FnBPA5‐poor parenchyma (Figure 4E), even though the mean FnBPA5 intensities were not statistically different between FnBPA5‐rich parenchyma and MVPs (Figure S7C, Supporting Information). Importantly, though, the highest CD45+/CD68+ macrophage infiltration was seen in the FnBPA5‐rich parenchyma (18 ± 9%), compared to MVPs (5 ± 4%) as well as the parenchyma that did not show the presence of untensed fibronectin fibers (8 ± 7%; Figure 4F).

### 2.5 Leukocytes Formed CD45+ Clusters in Proximity to ECM Rich in Disorganized Collagen Fibers

**Figure 5:**
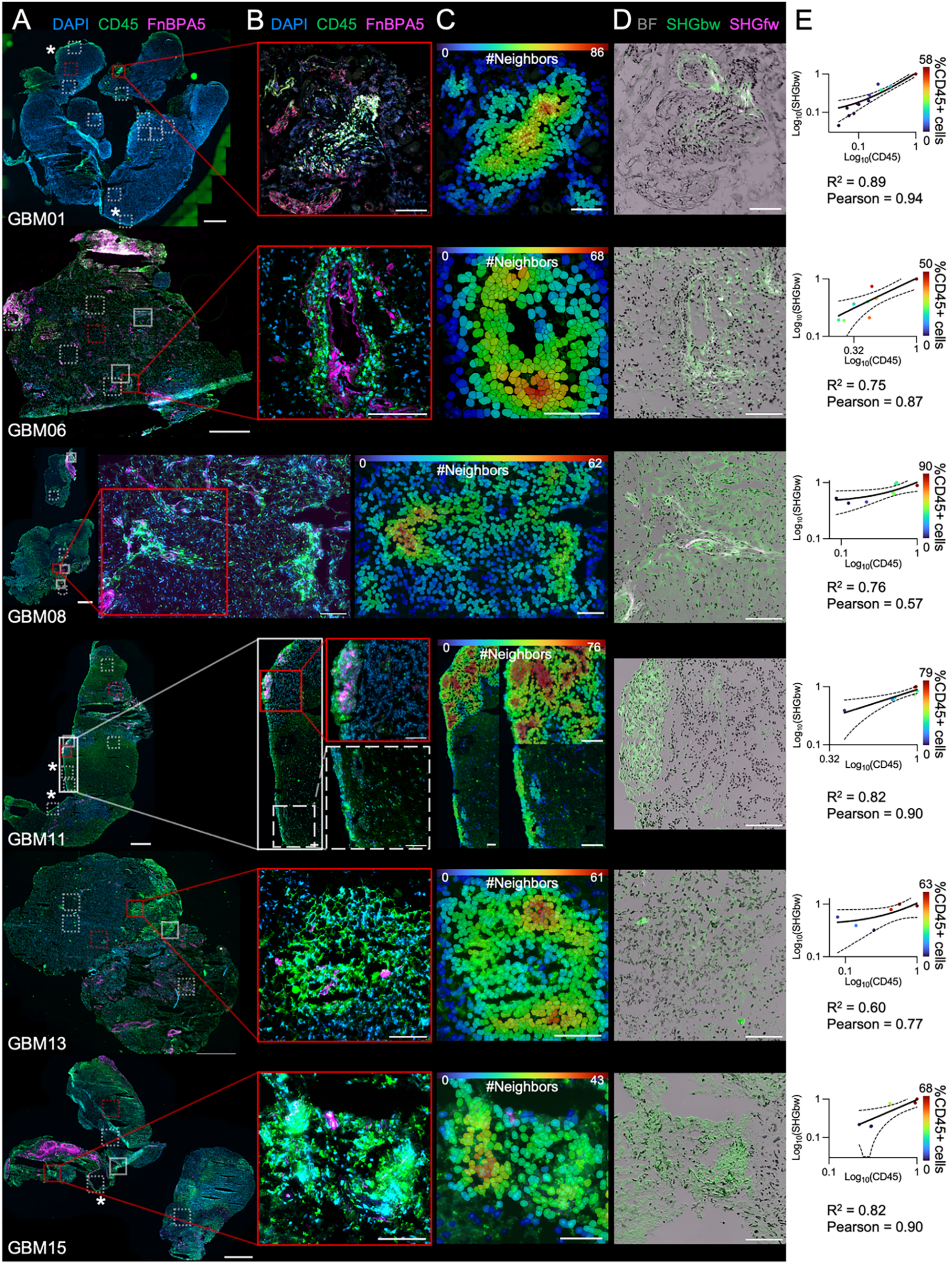
Leukocytes form CD45+ clusters in proximity to ECM rich in disorganized collagen fibers as probed by SHG. A) Representative image from glioblastoma whole tissue scans showing immunostaining for CD45 (green) and DAPI (blue). Only the samples showing CD45+ aggregates are shown. Non‐dotted squares highlight the CD45+ aggregates. Dotted squares indicate CD45+ areas and areas sampled for the graph in E). Red squares indicate representative areas reported in Figure S6 (Supporting Information). Bars are 1mm. B) Representative confocal images (CD45 green, FnBPA5 magenta, DAPI blue) of CD45+ aggregates. Bars are 100 µm. C) Cell shapes color‐coded according to the number of neighboring cells within 50 µm of their center show cluster formation. Bars are 100 µm. D) Representative brightfield (BF, gray) images overlayed with Second Harmonic Generation (SHG) signals in the backward (bw, green) and forward (fw, magenta) direction of the same field of views shown in C). Bars are 100 µm. E) Normalized CD45 intensity plotted against normalized SHGfw intensity. 1 dot is 1 FOV from A) and is color‐coded according to the percentage of CD45+ cells. Lines represent linear regression ± confidence intervals, R^2^ the goodness of fit and Pearson the Pearson R correlation coefficient. Additional data can be found in Figure S8 (Supporting Information).

While staining for macrophages, we observed CD45+ cell aggregate in 6/14 of the stained samples, as opposed to necrotic/perinecrotic areas that are rich in CD45 but poor in DAPI stainings (**Figure** **5**). N≥2 aggregates were found in all the samples, except for n = 1 in GBM01 (Figure 5A). CD45+ aggregates are highlighted by the non‐dotted squares in Figure 5A and representative ones are given in Figure 5B. To validate that these cells are organized as high‐density clusters, each cell was color‐coded according to the number of neighboring cells found within a radius of 50 µm (Figure 5C). This confirmed that leukocytes consistently formed organized clusters in several glioblastomas. Untensed fibronectin fibers were detected within CD45+ aggregates in 4/6 tumors (GBM01, GBM06, GBM08, GBM11; Figure 5B), which were also rich in SHG signal. SHG‐forward (SHGfw) detects big fibers, SHG‐backward (SHGbw) small, disorganized fibers^[^ ^30^ ^]^ (see Experimental Section). Intriguingly, all CD45+ aggregates showing SHGbw, formed discontinuous, non‐encapsulating patterns around the aggregate margins (Figure 5D). In the bulk of bigger clusters, SHGbw flanked cell nuclei (Figure 5D, green; GBM01, GBM06, GBM08, GBM11). This suggests that CD45+ aggregates are close to the more disorganized collagen fibers. While collagen I forms well‐organized fibrils, also collagen V can contribute to the SHG in glioblastoma, as it can co‐localize with collagen I/III thereby promoting the formation of smaller, less organized structures.^[^ ^90^ ^]^

In addition to observing densely aggregated CD45+ cells, we identified populations of CD45+ cells that were dispersed rather than clustered (highlighted by dotted squares in Figure 5A). Interestingly, in these regions, we observed a pronounced linear correlation between leukocyte density (CD45+) and areas of disorganized collagen fibers, as measured by second‐harmonic generation backward (SHGbw) scattering (Figure 5E). By contrast, forward SHG (SHGfw) signals—indicative of tightly packed collagen I/III fibers—were detected in half of the samples analyzed (3 out of 6: GBM01, GBM06, GBM08; see Figure 5D, magenta; and Figure S8, Supporting Information). These SHGfw signals consistently appeared adjacent to relaxed fibronectin fibers and colocalized with prominent CD45+ aggregates (Figure 5D). Additionally, streak‐like arrangements of CD45+ cells were occasionally observed at tumor boundaries, such as in GBM01 and GBM11 (asterisks in Figure 5A), mirroring previous findings.^[^ ^60^ ^]^ Taken together, our data suggest that in glioblastoma, CD45+ cell aggregates are closely associated with regions enriched in densely packed collagen I/III fibers and untensed fibronectin fibers. Conversely, individual or sparsely distributed CD45+ cells are typically located adjacent to more disorganized collagen fibers, which may thus include collagen V or other collagens that disrupt the tight ordering and bundling of collagen fibers.^[^ ^90^ ^]^ Furthermore, leukocyte infiltration shows a direct proportionality to the SHGbw signal within the extracellular matrix (ECM), indicating that greater leukocyte presence is linked to less organized collagen architecture.

### 2.6 Glioblastomas Contained Lymphatic‐Endothelial‐Like‐Cells that Co‐Localize with CD45+ Clusters with Resemblance to Tertiary Lymphoid Structures (TLS)

**Figure 6:**
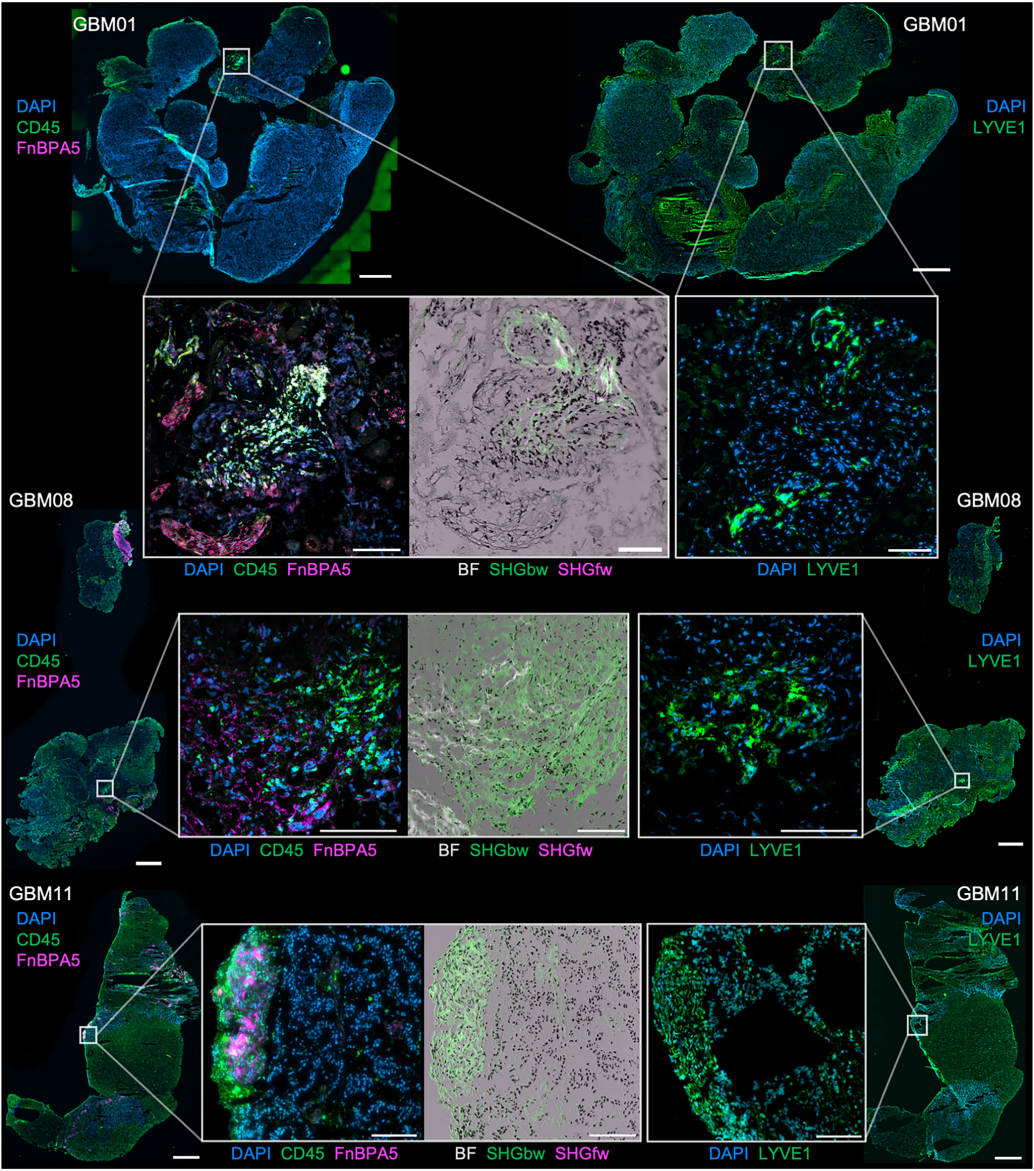
Glioblastomas contain lymphatic‐endothelial‐like (LEC‐like) cells that co‐localize with CD45+ clusters with resemblance to Tertiary Lymphoid Structures (TLS). 3/6 samples showed occasional CD45+ aggregates that co‐localized with LEC‐like cells as shown in this figure. Left whole tissue scans show CD45 (green), FnBPA5 (magenta) and DAPI (blue) with magnification of the field of views containing the aggregates near collagen I/III fibers (backward scattered SHGbw, green; forward scattered SHGfw, magenta) and untensed fibronectin fibers (FnBPA5). Right: whole tissue scans show Lymphatic Vessel Endothelial Hyaluronan Receptor 1 (LYVE1, green) and DAPI (blue) with magnification of the same field of views than on the left. Whole tissue scans stained for CD45‐FnBPA5 and LYVE1 belong to the same tumors (GBM01 top, GBM08 middle, GBM11 bottom) but show different tissue slices. Bars are 1 mm in the whole tissue scans, 100 µm in insets. Additional data can be found in Figure S8 (Supporting Information).

**Figure 7:**
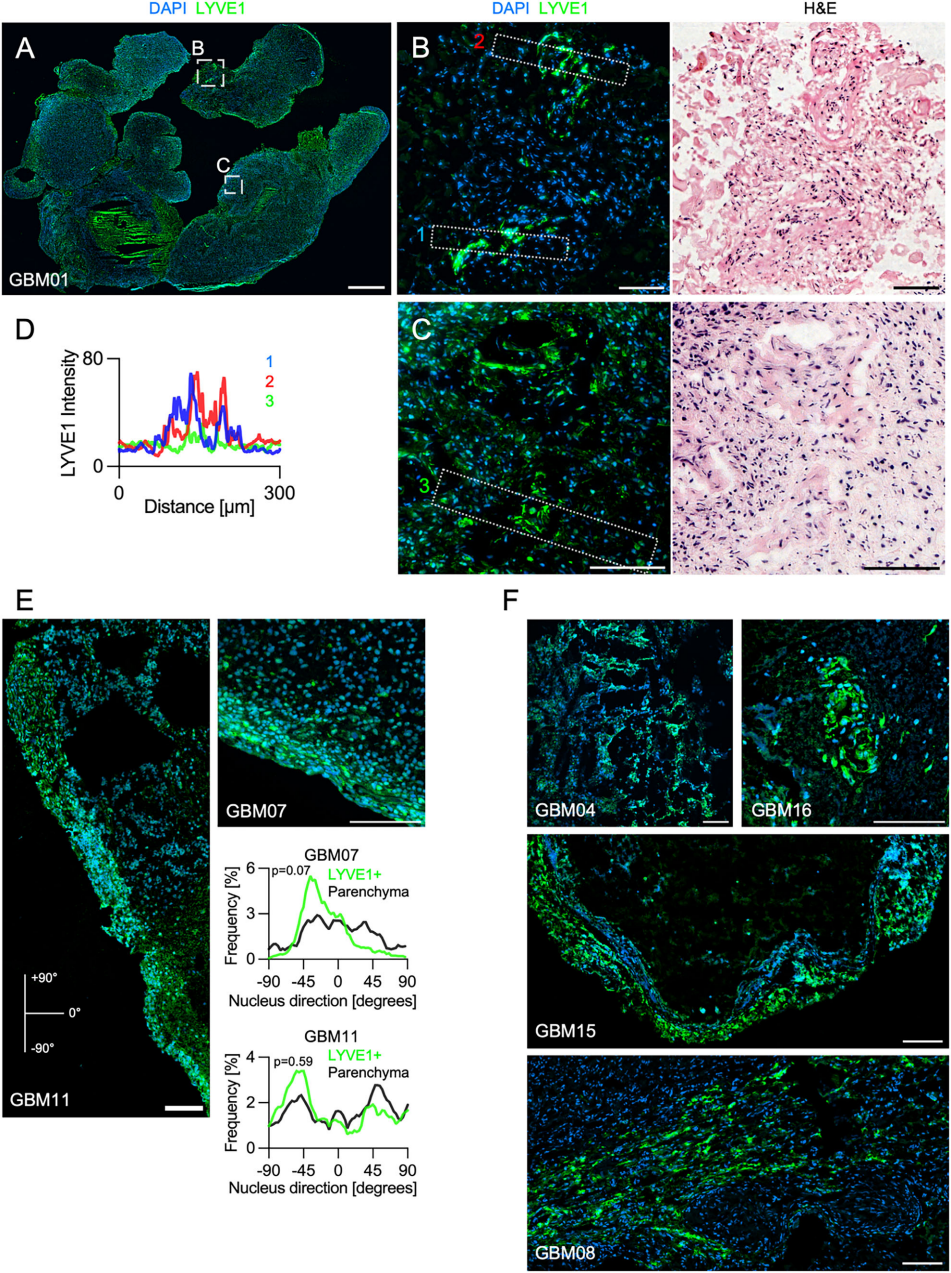
Glioblastomas contain lymphatic‐endothelial‐like (LEC‐like) cells that co‐localize with CD45+ clusters with resemblance to Tertiary Lymphoid Structures (TLS). A) Representative whole tissue image from GBM01 showing immunostaining for Lymphatic Vessel Endothelial Hyaluronan Receptor 1 (LYVE1, green) and DAPI (blue). The rectangles mark the areas represented in B,C. Bar is 1mm. B,C) Representative confocal B) and associated crop from slide‐scanner C) image of cells expressing LYVE1 in GBM01. Dotted rectangles enclose the 50 × 300 µm areas sampled for the line scans plotted in D). Bars are 100 µm. D) Intensity profiles (left to right) of the dotted rectangles as marked in B,C) and matched by color. E) Representative confocal images of cells expressing LYVE1 in the tumors GBM11 and GBM07. Bars are 100 µm. Graphs indicate the frequency distribution of the dominant direction of nuclear shapes sampled in LYVE1+ area and the parenchyma. Mann‐Whitney tests. Non‐statistically significant p‐values reported as 2‐digit numbers. F) Representative confocal images of cells expressing LYVE1 in the 4 remaining tumors expressing LEC‐like cells (GBM04, GBM16, GBM15, GBM08). Bars are 100 µm. Additional data can be found in Figure S9 (Supporting Information).

Growing attention is given to lymphatic‐mediated immunosurveillance in brain tumors,^[^ ^63^ ^]^ supported by the recent discovery that 54–81% of endothelial cells of patient‐derived glioblastoma express lymphatic endothelial cell markers and are thus called lymphatic‐endothelial‐like cells (LEC‐like).^[^ ^64^ ^]^ Furthermore, the formation of Tertiary Lymphoid Structures (TLS) was described in Glioblastoma.^[^ ^60^, ^61^ ^]^ TLS are “organized aggregates of immune cells that form postnatally in nonlymphoid tissues”.^[^ ^62^ ^]^ Unlike MVPs, TLS are non‐encapsulated by basement membrane. We thus asked whether structures similar to TLSs might be identified in our samples. In glioblastoma, TLS are rich in B‐ and T‐cells, as thoroughly characterized with CD20, CD4, CD8.^[^ ^60^ ^]^ As TLS can be preliminary defined with the pan leukocyte marker CD45,^[^ ^60^ ^]^ we asked whether there could be a link between the presence of CD45+ aggregates and the presence of LEC‐like cells, as assessed by LYVE1 immunostaining. Strikingly, we find here that LEC‐like cells co‐localized with the CD45+ aggregates, rich in collagen I/III and untensed fibronectin fibers, as identified in GBM01, GBM08 and GBM11 (**Figure** **6**). To ask if the expression of LEC‐like markers was consistent across several glioblastomas, we established a quantitative, imaging‐based criterion that assessed 50×300µm line scans (areas indicated with the dotted rectangles in **Figure** **7**B,C, profiles are in Figure 7D, see Experimental Section). We found that 7 out of 14 tumors contained LEC‐like cells (Figure S9, Supporting Information).

We also observed areas where some LYVE1+ cells arranged in streaks in GBM07 and GBM11 (Figure 7E). To further characterize these streaks, nuclear orientation analysis attested that they had their nuclei aligned along a dominant direction of orientation, unlike the neighboring parenchyma. Despite the distributions of nuclear orientation were not statistically different (Mann‐Whitney test), they peaked at around −30 and −45° (Figure 7E). Altogether, these results suggest that glioblastoma might be endowed with LEC‐like cells. Moreover, the remaining 4 tumors expressing LEC‐like cells (GBM04, GBM08, GBM15, GBM16) displayed LYVE1+ cells arranged as monolayers, not as mature lymphatic vessels (Figure 7F). Taken together, these results show that 50% (7/14) of our glioblastoma samples contained LEC‐like cells and were either associated with CD45+ aggregates or arranged as cell streams.

## 3 Discussion

As previous therapeutic strategies have failed to extend the survival of glioblastoma patients in clinical trials, the question arises whether fundamental knowledge in mechanobiological processes is lacking that could be exploited to improve on therapeutic options. While it is increasingly evident that biochemical and physical alterations of the tumor stroma drive not only stiffening but also modulate the tension of individual ECM fibers,^[^ ^65^, ^70^ ^]^ fibronectin expression and fibrillogenesis are consistently upregulated across cancer types, including in glioblastoma.

Leveraging a peptide probe capable of detecting fibronectin fiber tension‐as fiber stretching destroys its multivalent binding motive‐ our recent work has demonstrated that fibronectin fibers are highly tensed in healthy organs, but lose their tension in pathological ECM, as shown in both murine cancer and fibrosis models, as well as in human breast cancer.^[^ ^56^, ^65^, ^67^, ^68^, ^91^ ^]^ Given that the brain is the softest organ in the human body,^[^ ^35^, ^92^ ^]^ we specifically examined the remodeling of fibronectin fibers and how their tension might get altered in human glioblastoma cryosections. Notably, while the parenchymal ECM of healthy brain is enriched in GAGs, HA, CS, and various glycoproteins—but deficient in fibrillar collagen or Fibronectin—our peptide tension probe FnBPA5 revealed that fibronectin fibers are stretched in the basement membranes of MVs and MVPs, where they colocalize with collagen IV (Figure 3). In contrast, untensed fibronectin fibers are prevalently found in MVPs (Figures 1, 2; Figure S2, Supporting Information).^[^ ^21^ ^]^ Morphologically, MVPs are distinguished from MVs by their larger noncircular shapes and pronounced endothelial remodeling (Figures 2, 3). Unlike MVs, which display a single endothelial layer lining the luminal side of the basement membrane, as expected, MVPs contain endothelial cells that protrude into the vessel lumen and form stratified layers (see arrowheads in Figure 3D).

**Figure 8:**
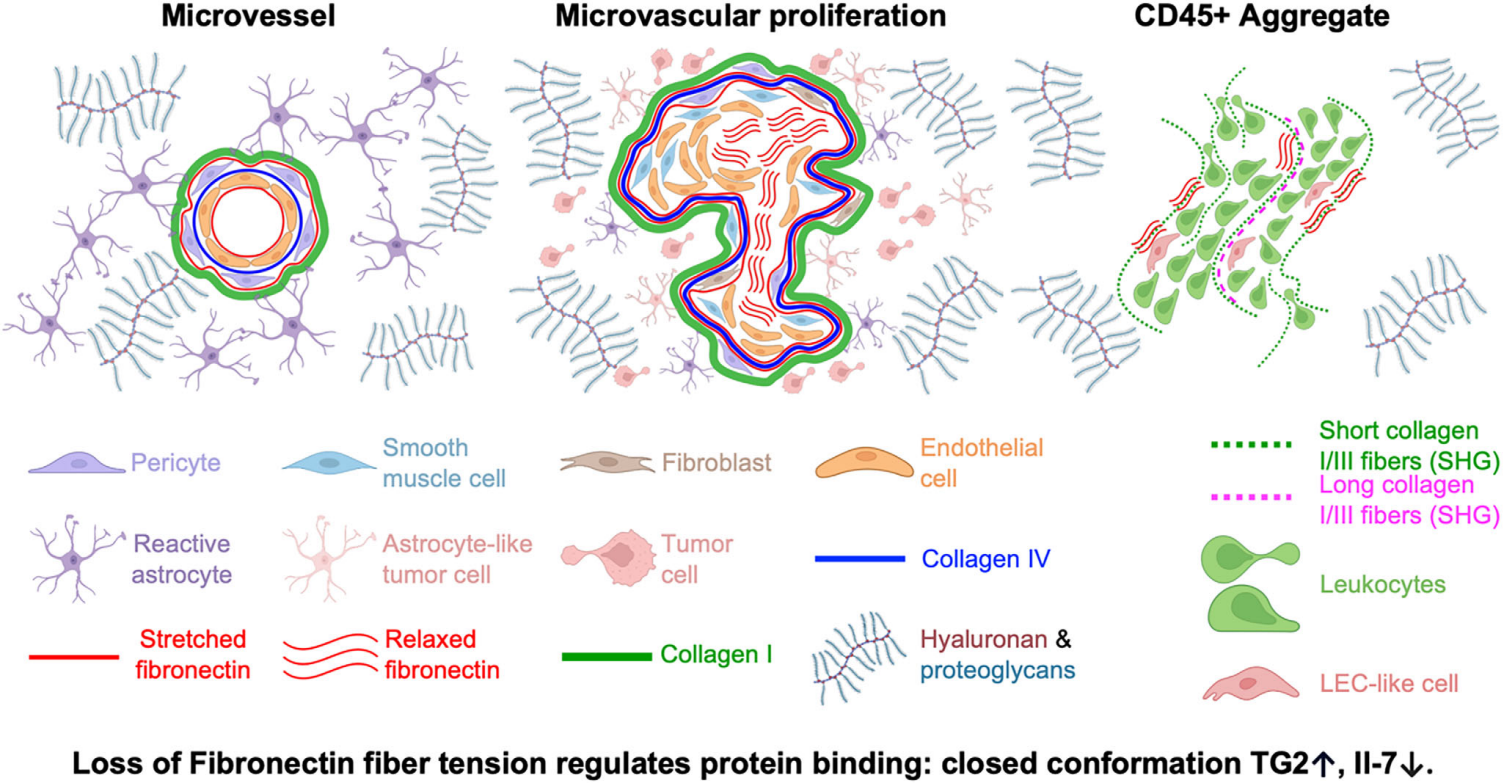
Summary sketch of morphological features of microvessels (MVs) in comparison to microvascular proliferations (MVPs). The collagen IV rich basement membranes of MVs and MVPs are rich stretched fibronectin fibers, with collagen I fibers forming an outer rim surrounding the vessel, rich in smooth muscle cells and pericytes. In the exterior surroundings, the ECM is rich in hyaluronan and proteoglycans. In MVs, astrocytes are known to form connections with the basement membranes and distribute homogeneously in space. This is not the case in MVPs, although they localize preferentially in their proximity, where neoplastic glial cells of astrocytic morphology are also found. MV lumens are devoid of untensed fibronectin fibers. Conversely, untensed fibronectin fibers are heavily enriched in the lumen of MVPs, where they are juxtaposed to stratified endothelial cells and α‐SMA expressing cells which could at least in part be activated fibroblasts that originate from occasional EndoMT events. Also, necrotic and non‐necrotic parenchyma areas rich in untensed fibronectin are found in glioblastoma tissues. Importantly, the density of untensed fibronectin fibers in the parenchyma correlates with the infiltration of macrophages. Finally, parenchymal areas with CD45+ aggregate contain organized clusters of leukocytes together with lymphatic‐endothelial‐like (LEC‐like) cells. In these regions rich in untensed fibronectin fibers, the ECM also contains short (green line) and long (magenta dashed line) collagen I/III fibers (green dashed line) and, occasionally, by collagen I/III long fibers (magenta dashed line). As several therapeutic approaches to treat glioblastoma are recently focusing on TG2 inhibitors, or on boosting IL‐7 signalling,^[^ ^105^, ^106^ ^]^ please note that untensed fibronectin fibers promote the binding of TG2 in its closed conformation,^[^ ^75^ ^]^ while stretched fibronectin enhances the binding of IL‐7.^[^ ^76^ ^]^

Our observations, as summarized in **Figure** **8**, are consistent with the histological definition of MVPs, as being rich in proliferative endothelial cells contributing to a multilayered endothelium adjacent to smooth muscle cells/pericytes and fibroblasts.^[^ ^2^, ^19^, ^24^, ^29^, ^40^, ^41^, ^86^ ^]^ Significantly, we found that MVPs not only harbor highly contractile, α‐SMA‐expressing cells within their lumens (Figure 1)‐alongside proliferative endothelial strata (Figure 3)‐ but also are enriched in untensed fibronectin fibers (Figure 2). This finding is functionally relevant: untensed fibronectin selectively engages α5β1 integrins, whereas fiber stretching increases the RGD loop‐synergy site distance, thus switching Fibronectin's preference to other RGD‐binding integrins.^[^ ^71^, ^73^, ^77^ ^]^ Given that α5β1 integrin activation robustly upregulates cell proliferation, we propose that activation of α5β1 integrin in response to untensed fibronectin may help to drive the high mitotic activity seen in MVPs‐ a hallmark of vascular hyperplasia. Thus, the emergence of untensed fibronectin fibers may be a key mechanistic driver of vessel remodeling and by their enlargement, rapid disease progression, although further experimental validation is required.

The mechanisms underlying MVP formation and progression remain poorly understood, but our detection of α‐SMA+ cells within MVPs (Figures 1, 2, 3) may reflect focal occurrences of endothelial‐mesenchymal transitions (EndoMTs). While pericytes wrap around the endothelial cells that line the capillaries and together assemble the basement membrane,^[^ ^43^ ^]^ the collagen I rim observed here around MV and MVPs may derive from pericytes or perivascular fibroblasts, even though little is known how CNS fibroblasts contribute to shaping the diseased microenvironment of MVPs.^[^ ^42^ ^]^ Outside of the basement membrane, we observe that MVs retain a network of GFAP+ cells (Figure S1D,E, Supporting Information) with GFAP filaments in close proximity to MVs (Figure 1C, arrowheads; Figure S1D, Supporting Information). These likely represent astrocytic end‐feet characteristic of a healthy blood‐brain barrier.^[^ ^37^, ^85^ ^]^ In glioblastoma, GFAP is expressed by both reactive astrocytes and neoplastic glial cells of astrocytic morphology,^[^ ^37^, ^39^ ^]^ but such structures are absent near MVP basement membranes (Figure 1C; Figure S1D, Supporting Information). This raises the possibility that at least a considerable fraction of GFAP+ cells are hyperproliferative neoplastic cells capable of generating hypoxic microenvironments.^[^ ^21^, ^25^ ^]^ Hypoxia is known to upregulate the expression of angiopoietins, TGF‐α/β, TNF‐α and VEGF,^[^ ^26^, ^27^ ^]^ which can induce necrosis as seen in various tissue sections (Figure S2, Supporting Information), mesenchymal transitions,^[^ ^93^ ^]^ or drive angiogenesis as well as the formation of MVPs.^[^ ^25^, ^26^, ^27^ ^]^ Angiopoietins also promote mesenchymal transitions of a variety of specialized cells^[^ ^94^ ^]^ and cell–cell interaction analyses predict substantial endothelial‐to‐perivascular cell ligand–receptor cross‐talk.^[^ ^95^ ^]^ Transcriptomic profiling via RNA‐seq has demonstrated that endothelial cells in GBM microenvironments undergo mesenchymal transformation and stemness‐like activation^[^ ^96^ ^]^ Emerging evidence suggests that EndoMT is spatially restricted to discrete clusters of endothelial cells,^[^ ^97^, ^98^ ^]^ facilitating aberrant vascularization.^[^ ^95^ ^]^ Thus, it is plausible that MVPs act as localized hotspots where EndoMT events might have occurred—a possibility with significant therapeutic implications, as mesenchymal phenotype acquisition confers resistance to radio‐, chemo‐ and immunotherapies.^[^ ^97^, ^99^, ^100^ ^]^

The MVP microenvironment is highly heterogeneous and frequently infiltrated by immune cells.^[^ ^8^, ^28^, ^55^, ^59^, ^85^, ^87^ ^]^ Our data indicate that macrophages are more densely distributed in parenchymal ECM that is enriched with untensed, randomly oriented fibronectin fibers than in MVPs or parenchyma with sparse untensed fibronectin fibers (Figure 4). While the causality of this association remains to be elucidated, prior work suggests that fibronectin fiber relaxation may follow neutrophil infiltration in acute inflammation models.^[^ ^91^ ^]^ In glioblastoma, recent findings indicate that endothelial cells interact strongly with monocyte‐derived macrophages (regardless of M1/M2 status) infiltrating from the vasculature, but not with resident microglia.^[^ ^87^ ^]^ Thus, CD45+/CD68+ macrophages adjacent to untensed fibronectin may include microglia. Additionally, we observed CD45+ leukocyte aggregates near lymphatic‐endothelial‐like (LEC‐like) cells in 3 out of 6 glioblastoma samples, typically within or near collagen I/III‐rich ECM containing untensed fibronectin, both in the parenchyma and at the tumor margin (Figure 5). These aggregates resemble tertiary lymphoid structures (TLS) described in glioblastomas and other cancer types.^[^ ^60^, ^61^, ^62^ ^]^ TLSs facilitate the invasion of immune cells into tumor sites and have therefore attracted significant attention for therapeutic manipulation as a means of improving anticancer immunity and favorable treatment response in patients.^[^ ^60^, ^61^, ^62^ ^]^ As LEC‐like cells co‐localize with CD45+ aggregates, VEGF‐C from LEC‐like cells may drive lymph angiogenesis and facilitate immune cell circulation.^[^ ^63^ ^]^

Complementing the prevailing assumption that densely packed, aligned collagen fiber bundles around tumor nests act as a physical barrier to anti‐tumor immunity (as in melanoma),^[^ ^57^, ^58^, ^101^ ^]^ we found that leukocyte infiltration correlates directly with less organized collagen within the ECM, as revealed by SHG imaging. Collagen V is implicated in the disorganization of collagen fibers^[^ ^90^ ^]^ and is associated with the mesenchymal subtype and tumor invasiveness.^[^ ^102^ ^]^ CD45+ cell clusters are frequently located next to collagen I/III fibers, while the more sparsely distributed CD45+ cells are associated with these more disorganized collagen fibers, potentially enriched in collagen V. Leukocytes also express high levels of GPR56, which promotes immune cell migration and binds collagen III and TG2; this interaction is upregulated in GBM.^[^ ^103^, ^104^ ^]^ This adhesion GPR is known to bind collagen III as well as TG2, whereby their binding sites on GPR56 overlap, and GPR56 mRNA is upregulated in GBM and other astrocytomas in comparison to normal brain samples.^[^ ^103^ ^]^ While GPR56 might thus be involved in promoting the here observed infiltration of CD45+/CD68+ macrophages and of CD45+ leukocytes into regions rich in collagen fibers (Figure 5; Figure S8, Supporting Information), ECM tethering of TG2 might enrich its local concentration. TG2 in its closed conformation has higher affinity to fibronectin when its fibers have lost their tension, either via proteolytic cleavage our due to collagen fibers taking over as force‐bearing elements.^[^ ^75^ ^]^ Our data show that enhanced ECM binding of TG2 in regions enriched in untensed fibronectin fibers, namely in ‐MVPs, is not observed though (Figure S10, Supporting Information), suggesting that TG2 may exist primarily in its open, active state, which is unaffected by fiber strain.^[^ ^75^ ^]^ Multiple independent studies corroborate that active TG2 is enriched in glioblastoma.^[^ ^79^, ^105^ ^]^

In summary, our study identifies the presence of untensed fibronectin fibers as a distinctive feature of glioblastoma, particularly within MVPs and immune cell‐rich microenvironments where they co‐localize with collagen I/III. Although our sample size of biopsy tissues is finite, we propose that mechanical regulation of fibronectin fiber tension is a critical, yet underappreciated, factor in glioblastoma progression. This is noteworthy because several Fibronectin‐binding interactions—including those of TG2 and IL‐7, both of which exhibit mechano‐regulated affinities—are currently under investigation for the therapeutic targeting in glioblastoma.^[^ ^105^, ^106^ ^]^ Inhibition of TG2 is gaining traction due to its role in mesenchymal transition and tumor aggressiveness,^[^ ^78^, ^79^, ^80^ ^]^ while IL‐7‐based immunotherapies are demonstrating efficacy in preclinical models.^[^ ^107^, ^108^, ^109^ ^]^ As shown previously, the closed conformation of TG2 preferentially binds untensed fibronectin and, upon activation, promotes tumor progression,^[^ ^75^, ^79^, ^105^ ^]^ while IL‐7 binds stretched fibronectin fibers with higher affinity and supports anti‐tumor immunity.^[^ ^76^ ^]^ These opposing, tension‐dependent effects imply that the ECM's mechanical state could be a pivotal determinant of glioblastoma outcomes, a hypothesis that we hope will stimulate further in‐depth investigations.

Our results indicate the potential to harness peptides with mechano‐regulated affinities—such as radiolabeled FnBPA5—for the diagnosis and therapeutic targeting of abnormal ECM in glioblastoma. Prior studies have shown that FnBPA5 can serve in animal models as a fibronectin‐targeting radiotracer for the SPECT/CT imaging of prostate cancer.^[^ ^68^, ^110^ ^]^ Thus, future strategies might introduce mechano‐regulated interventions, such as FnBPA5‐drug conjugates, to selectively target the mechanically altered ECM in MVPs. We thus propose that mechanical alterations of ECM fibers‐here demonstrated for fibronectin‐ should be considered an integral factor in glioblastoma progression and therapy design. Discovering that fibronectin fibers lose their tension in MVPs and in immune cell‐infiltrated areas, is significant as ECM fiber tension, and thus their physical state, can tune molecular interactions. Physical state‐specific drug targeting was not previously explored to inhibit tumor growth or boost immunotherapies. While initial insights are provided here, the complex relationships between fibronectin fiber tension, the regulation of ECM storage or activity of fibronectin‐binding partners, and their potential role in modulating glioblastoma progression requires further investigations. Further in vivo animal studies are needed to test and validate these hypotheses. Mechano‐targeted interventions, including the use of fibronectin‐binding drug conjugates or imaging tracers like FnBPA5,^[^ ^68^, ^110^ ^]^ may offer novel strategies to selectively target the tumor microenvironment and enhance the effectiveness of immunotherapies.

## 4 Experimental Section

### Human Brain Tumor Biopsy Sampling and Characterization

Tumor tissues from human brain were collected from patients by the Neuropathology Department of University Hospital Zurich (USZ) and the Tissue Bank Bern (TBB) and de‐identified in accordance with the Business Administration System for Ethics Committees (BASEC) guidelines (ethical approval “Mechanobiology of Extracellular Matrix” BASEC‐Nr. 2017‐01828). Informed written consent was obtained from each patient prior to surgical resection of brain tissue. Technicians and neuropathologists from the Neuropathology Department of USZ, as well as pathologists from TBB, prepared cryosections following macroscopic and microscopic diagnosis. These sections were banked and stored at −80 °C until needed. Further characterization of the resected tissues, as detailed in Table 1, was conducted by pathologists from USZ and TBB.

### Immunohistochemistry of Human Patient Tissue Cryosections

Cryopreserved tissue sections were stained by us for specific ECM markers and the FnBPA5‐Cy5/scrFnBPA5‐Cy5 tension probe. Briefly, 5 µm thick cryosections were thawed at room temperature for 10 min, rehydrated and rinsed with Phosphate‐Buffered Saline, Dulbecco's formula, (DPBS), then blocked for 30 min with 4% bovine serum albumin (BSA). For experiments involving FnBPA5, tissues were incubated for 1 h at room temperature with 5 µg mL^−1^ of FnBPA5‐Cy5 and washed three‐times with DPBS (5min per wash). The sections were subsequently fixed with 4% paraformaldehyde (PFA) in 1xPBS for 10 min and washed thrice with Tris‐buffered saline (TBS, 5min per wash), permeabilized with 0.1% Triton X‐100 in 1X DPBS for 30 min, washed three‐times with TBS (5min per wash). The tissue sections were then blocked with Animal‐Free Diluent RT (VectorLabs Nr. SP5035) supplemented with 0.01% Triton X‐100 for 45 min, followed by overnight incubation at 4 °C with the primary antibodies (**Table** **2**) diluted in Animal‐Free Diluent RT (VectorLabs Nr. SP5035). The next day, tissues were washed three‐times in TBS. Secondary antibodies (Table 2) were diluted in Animal‐Free Diluent RT (VectorLabs Nr. SP5035) and applied for 1 h at room temperature followed by 3 washing steps in TBS (5min/wash). DAPI stain (2 µg mL^−1^, 5 min) was then performed, followed by 3 washing steps in TBS (5min/wash). Finally, stained tissues were mounted with ProLong Gold Antifade Mountant (ThermoFisher Scientific, Nr. P36930) and left 48 h at room temperature before imaging.

**Table 2:** List of antibodies used throughout this work.

Antigen	Host	Company	Reference	Dilution
Fibronectin	Rabbit polyclonal	Abcam	AB23750	1:100
CD31	Mouse monoclonal	Abcam	AB9848	1:100
Collagen I	Rabbit polyclonal	Abcam	AB34710	1:100
Collagen IV	Mouse monoclonal	Abcam	AB6311	1:100
GFAP	Mouse monoclonal	Biolegend	644 701	1:100
α‐SMA	Goat polyclonal	ThermoFisher	PA5‐18292	1:100
CD45	Rabbit monoclonal	Abcam	AB40763	1:100
CD68	Mouse monoclonal	Abcam	AB955	1:200
LYVE1	Rabbit monoclonal	Abcam	AB219556	1:100
TG2	Mouse monoclonal	Abcam	AB2386	1:100
anti‐Rabbit IgG, Alexa Fluor 488	Goat polyclonal	ThermoFisher	A11034	1:200
anti‐Mouse IgG, Alexa Fluor 555	Goat polyclonal	ThermoFisher	A21424	1:200
anti‐Mouse IgG, Alexa Fluor 488	Donkey polyclonal	ThermoFisher	A21202	1:200
anti‐Rabbit IgG, Alexa Fluor 546	Donkey polyclonal	ThermoFisher	A10040	1:200
anti‐Goat IgG, Alexa Fluor 633	Donkey polyclonal	ThermoFisher	A21082	1:200

### Imaging

Full tissue imaging of stained cryosections of tumor tissues was performed with a slide scanner microscope (Panoramic 250, 3D Histec; 20X objective). 4‐channel images were converted from.mrxs to.ometiff format using NGFF‐converter (Glencoe software). High‐resolution confocal images were obtained with a Leica SP8 confocal microscope using a 40X water‐immersion objective. For correlative SHG imaging, mounted tissues were incubated for more than 24 h in DPBS at room temperature, their coverslips removed, stained for hematoxylin and eosin (H&E) following a standard protocol implemented on a linear stainer COT20 (Medite). Tissues were then mounted with Pertex mounting medium (Histolab Nr 00811) and imaged on a Leica SP8 multiphoton microscope (25X water‐immersion objective) equipped with an InSight laser (880 nm excitation wavelength) and a dual‐detector configuration for backward and forward second harmonic signal sampling. SHG was sampled in the forward (SHGfw) and backward (SHGbw) directions, where SHGfw is generally associated with bigger ECM fibers (in the order of magnitude of the wavelength λ = 880nm along the axial direction) and SHGbw with smaller, more randomly oriented ones (around λ/2).^[^ ^30^ ^]^ SHG was sampled in at least 3 non‐necrotic CD45+ Fields of View (FOV) and 3 non‐necrotic CD45‐ FOVs in all the tumors where we detected CD45+ aggregates. For SHG‐COL1A1‐COL4A1‐FnBPA5 acquisitions no DAPI stain was used and single confocal slices were obtained. Image acquisition parameters and look‐up‐tables in all the figures were always identical for MVPs and MVs belonging to the same sample.

### Image Analysis

Images were visualized and analyzed with Fiji (ImageJ), QuPath^[^ ^111^ ^]^ or MATLAB (Mathworks, Switzerland) as described below.

### Vessel Isolation from Whole Tissue Scans and Confocal Images

For the morphometric and signal analysis of vessels, we isolated from whole tissue images (Figure 2; Figure S3F–J, Supporting Information) and confocal FOVs (Figures 2, 3; Figure S6, Supporting Information) single structures based on the superposition of Fibronectin‐CD31‐FnBPA5 signals utilizing a custom macro in Imagej/Fiji that sampled vessels based on a threshold. Morphometric analysis were carried out on each vessel, the data from GBM01 plotted as Cumulative Distribution Functions in Figure 2F,G. When sampling scrFnBPA5 mean values, differentiation between MVP and MV was performed by morphometrical classification of vessels by circularity and area (MV: circularity > 0.6, area < 635 µm^2^; MVP: circularity < 0.5, area > 5406 µm^2^; numbers are the mean values in Figure 2H,I).

Data from all the tumors were then averaged and plotted as boxplots In all the boxplots, 1 dot represents the average from 1 patient sample and the bigger, black dot is the mean value (color‐coding as in Figure 1E is uniform throughout this work).

### Line Profile Plots and Pixel Analysis

Line profile plots of vessels and lymphatic endothelial cell marker were created using Fiji's built‐in “plot profile” function and then transferred to GraphPad Prism for visualization. For pixel analysis in Figure 3G we utilized a custom‐written Imagej/Fiji macro that isolated CD31/FnBPA5 positive pixels (confocal images) within each vessel based on a threshold. Results were then correlated for the two channels.

### Cross‐Correlation Analysis

Cross‐correlation analysis were performed on MATLAB by applying the following function

C = normxcorr2(Channel1, Channel2)

That computes the normalized cross‐correlation between two images. The maximum of the cross‐correlation matrix was considered as final value. Each vessel (confocal images) was previously isolated from the image, the channels split and the signal outside its boundary set to 0.

### Single‐Cell Analysis and Classification

Single cell detection was performed with QuPath 0.4.2 utilizing the built‐in function “cell detection”, which identified cell shapes based on the DAPI signal. For Figures 1, 4, confocal images (2 FOVs per condition per tumor sample) were used. For distance analysis in Figure 1, all the vessels were carefully annotated based on the collagen I signal. Then, the built‐in QuPath function “spatial analysis > distance to annotations 2D” allowed to retrieve the distance between each cell and the collagen I annotation, whereas the cells positive for a marker were identified with the built‐in QuPath function “Create single measurement classifier”, which classified single cells based on a signal intensity sampled within their shape. We calculated the intensity distributions in the surrounding of collagen I annotations (Figure 1F; Figure S1B, Supporting Information) with a custom‐written Imagej/Fiji macro that allowed us to sample intensity values by sequentially increasing the boundary of collagen I annotations by 1 µm steps (1 to 25 µm; vessel cross‐section was excluded from sampling; trends were normalized with respect to the value at the first step). To determine CD45+/CD68+ macrophages (Figure 4), we used a composite classifier, which is built by applying the logic operator “AND” to the 2 single measurement classifiers. Here we considered the whole FOVs as reported in Figure 4. CD45+ cells in Figure 5 were identified with the same method from whole tissue images, where the FOV corresponding to the SHG FOV was carefully annotated on the whole tissue image to match its size (Figure 5A indicates all the FOVs).

### Fiber Analysis and Orientation Analysis

For fiber analysis in Figure 4, we applied the built‐in Fiji function “Frangi Vesselness” (parameters: number of scales = 1, minimum = 0.3, maximum = 100″).^[^ ^112^ ^]^ The functions “Distribution of orientations”^[^ ^113^ ^]^ and “OrientationJ dominant direction”^[^ ^114^ ^]^ from the OrientationJ plugin suite were utilized to compute coherency. A custom‐written Imagej/Fiji macro allowed us to binarize images obtained with “Frangi Vesselness” function and to compute their number and size.

### CD45+ Cell Aggregates and SHG Analysis

CD45+ cell aggregates by identifying areas rich in DAPI and CD45 signal from whole‐tissue images are visually identified. To calculate the number of neighboring cells within a radius of 50 µm from each cell, the functions “cell detection” are applied and then “Calculate features > Add smoothed features (radius 50 µm)” in QuPath. The number of neighboring cells within a radius of 50 um was visualized in Figure 5C with the function “Show measurement maps” in QuPath. Correlation plots between SHGbw and CD45 intensity were obtained by sampling CD45 mean values from the FOVs indicated in Figure 5A (n > 6 per tissue slide, whole tissue images) and matched with the corresponding multiphoton image.

### Determination of Glioblastomas Expressing LEC‐Like Cells and Nuclear Orientation Analysis

To determine which samples expressed LEC‐like cells, 3 areas per glioblastoma sample with 50 × 300µm line scans (Figure 6B,C; Figure S9C, Supporting Information) were examined. LYVE1+ areas were considered those where the maximum of the line‐scan trend was greater than twice the baseline level. Glioblastomas with all the 3 trends fulfilling this criterion were considered expressing LEC‐like cells. Figure S9C (Supporting Information) indicate areas sampled with line scans.

Nuclear orientation analysis in Figure 7 was performed in Fiji on confocal images with the plugin StarDist,^[^ ^115^ ^]^ which allowed us to retrieve single nuclear shapes. The function “OrientationJ dominant direction” was then applied to each nucleus with a custom‐written macro and their distribution plotted in GraphPad Prism for visualization.

### Plot Generation

Boxplot were generated in Rstudio with ggplot package. All the other plots were generated in GraphPad Prism 10.0.

### Statistical Analysis

All the statistics were performed in GraphPad Prism 10.0. Stars in graphs indicate P values as follows: ^∗^ *p* < 0.1, ^∗∗^ *p* < 0.01, ^∗∗∗^ *p* < 0.001, and ^∗∗∗∗^ *p* < 0.0001 – non‐statistically significant *p*‐values are reported as 2‐digit numbers. In all the boxplots, data are presented with mean ± SD; 1 dot represents the average from 1 patient sample; bigger, black dots represent the mean value; the thick horizontal line represent median value. Here follows a figure‐by‐figure breakdown of statistical tests and sample size utilized to compare statistical significance or correlation between groups (details are in figure legends):

- Figure 1: one‐tailed unpaired t‐tests; n = 11;
- Figure 2: Kolmogorov‐Smirnov tests for CDF in GBM01, one‐tailed unpaired t‐tests for groups; n = 16;
- Figure 3: Spearman's test for CD31‐FnBPA5 trends, one‐tailed unpaired t‐tests for groups, linear regression for FnBPA5‐CD31 positive pixels; n = 9;
- Figure 4: RM one‐way ANOVA test with Geisser‐Greenhouse correction and Turkey's multiple comparison test when having triplets, one‐tailed unpaired t‐tests for couples; n = 9;
- Figure 5: Linear regression and Pearson's test for each trend (1 trend per sample);
- Figure 7: Mann‐Whitney tests for distributions of nuclear direction.

Statistical details of experiments (significance of the error bars and statistical test applied) can be found in the legends of each figure. Power of statistically significant tests (i.e., the probability from 0 to 1 that the number of samples correctly identifies a true effect) is reported in figure legends as “pwr” and was computed with MATLAB as follows: pwr = sampsizepwr(“t2”, [Mean1, SD1], Mean2, [], n), where Mean1 and SD1 are the mean and standard deviation of the tensed fibronectin fibers group, Mean2 is the mean of the untensed fibronectin fibers group, n the number of patients. In Figure 4, Mean2 is the MVP group.

## Conflict of Interest

VV is a co‐founder of the ETH start‐up Company Tandem Therapeutics (CH020.3.053.146‐7). The other authors declare no competing interests.

## Author Contributions

M.C. performed data curation, conceptualization, formal analysis, investigation, visualization, initiation of collaboration with TM at TBB and with TH at USZ, wrote and performed ethical approval amendment, wrote the original draft, wrote, reviewed, and edited the manuscript; I.G. performed project initiation, investigation, methodology, initiation of collaboration with KF, wrote – ethical approval amendment; A.M. performed investigation, wrote, reviewed, and edited the manuscript; K.F. performed project initiation, methodology, tissue diagnostics, providing insights and inputs into the pathological aspect and correlation to histopathological findings; T.M. performed methodology, tissue diagnostics, providing insights and inputs into the pathological aspect and correlation to histopathological findings; T.H. performed methodology, tissue diagnostics, providing insights and inputs into the pathological aspect and correlation to histopathological findings; V.V. performed conceptualization, supervision, funding acquisition, formal analysis, initiation collaboration with KF, wrote and received ethical approval, wrote study protocol, wrote, reviewed, and edited the manuscript. All authors have seen and approved the manuscript.

## Supporting information



Supporting Information

## Data Availability

The data that support the findings of this study are available on request from the corresponding author. The data are not publicly available due to privacy or ethical restrictions.
