# Supplementary material for: Loss of Fibronectin Fiber Tension in Glioblastoma is Associated with Microvascular Proliferations and Immune Cell Infiltration
Source: Adv Sci (Weinh). 2025 Sep 19;12(45):e16526. doi: 10.1002/advs.202416526 (PMC12677643; doi:10.1002/advs.202416526)
Supplement: Supplementary file 1 — Supporting Information [file ADVS-12-e16526-s001.docx]

**SUPPORTING INFORMATION**

**Loss of Fibronectin Fiber Tension in Glioblastoma is Associated with Microvascular Proliferations and Immune Cell Infiltration**

Michele Crestani^1^, Isabel Gerber^1^, Arnaud Mieville^1^, Katrin Frauenknecht^2^, Theoni Maragkou^3^, Tibor Hortobagyi^4^, Viola Vogel^1 *^

^1^ Laboratory of Applied Mechanobiology, Department of Health Sciences and Technology, ETH Zurich, Switzerland

^2^ National Center of Pathology (NCP), Laboratoire National de Santé (LNS), Luxembourg

^3^ Institute of Tissue Medicine and Pathology, University of Bern, Bern, Switzerland

^4^ Department of Neuropathology, University Hospital Zurich, Switzerland

^*^ Corresponding author: Viola Vogel ([viola.vogel@hest.ethz.ch](mailto:viola.vogel@hest.ethz.ch))

**List of Supporting Information**

- Figure S1 (data related to Figure 1)
- Figure S2 (data related to Figure 2)
- Figure S3 (data related to Figure 2)
- Figure S4 (data related to Figure 2)
- Figure S5 (data related to Figure 2)
- Figure S6 (data related to Figure 3)
- Figure S7 (data related to Figure 4)
- Figure S8 (data related to Figures 5-6)
- Figure S9 (data related to Figure 7)
- Figure S10


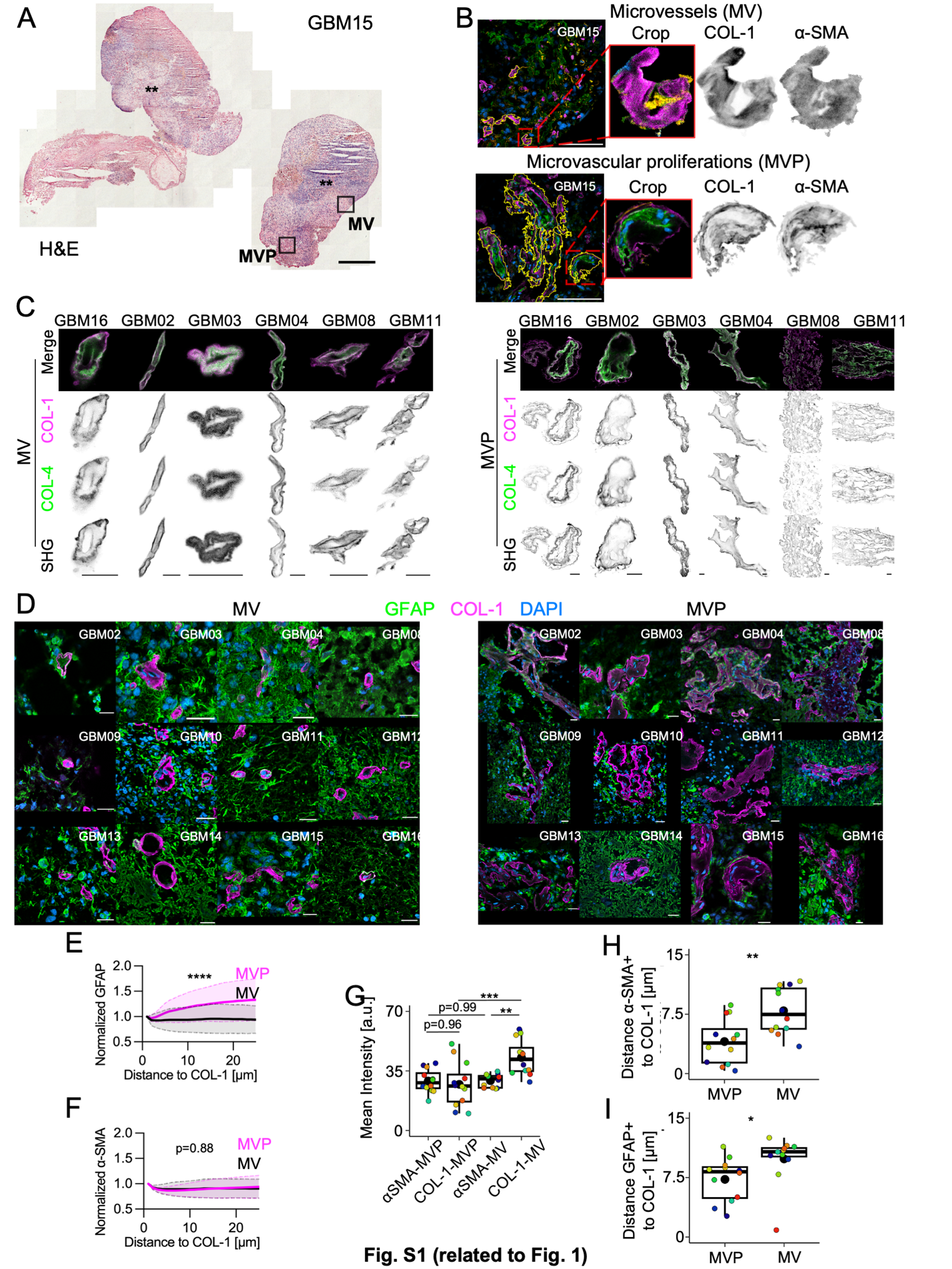


**Figure S1 (data related to Figure 1). (A)** Representative Hematoxylin and Eosin (H&E) image from GBM15 whole tissue obtained a posteriori from the same sample displayed in Fig 1A. Asterisks indicate areas with edema or necrotic that are negative for GFAP and α-SMA in Figure 1A. Bar is 1mm. In A and B, squares highlight representative microvascular proliferations (MVP) and microvessels (MV) shown in Figure 1B, C. **(B)** Representative image highlighting the detection (yellow lines), crops and channel separation on MVP and MV. Bar is 100 µm. **(C)** Representative MV and MVP crops from single confocal slices of samples stained for collagen I (magenta) and collagen IV (green), plus Second Harmonic Generation (SHG) signal obtained from 6 selected glioblastomas. Bars are 20 µm. **(D)** Confocal image crops showing the surroundings of representative MV and MVPs over 12 glioblastoma samples (GFAP green, Collagen I magenta, DAPI blue). Connections between GFAP filaments are more frequent in MV than MVP. MVP often have the external areas adjacent to collagen I devoid of GFAP. Bars are 20 µm. **(E, F)** GFAP **(E)** and α-SMA **(F)** intensity trend over distance from collagen I margin, normalized over the value obtained at distance = 0 µm. Intensity was sampled over rings increasing with 1µm steps (0 to 25 µm) and only the area external to the collagen I border was sampled. Dashed lines represent the standard deviation. Mann-Whitney test. **(G)** Mean intensity of α-SMA and collagen I signal within MVP and MV borders. Ordinary one-way ANOVA test with Turkey’s multiple comparison test. **(H, I)** Mean distance of α-SMA+ **(H)** and GFAP+ **(I)** cells from the nearest collagen I border. One-tailed unpaired t-tests.


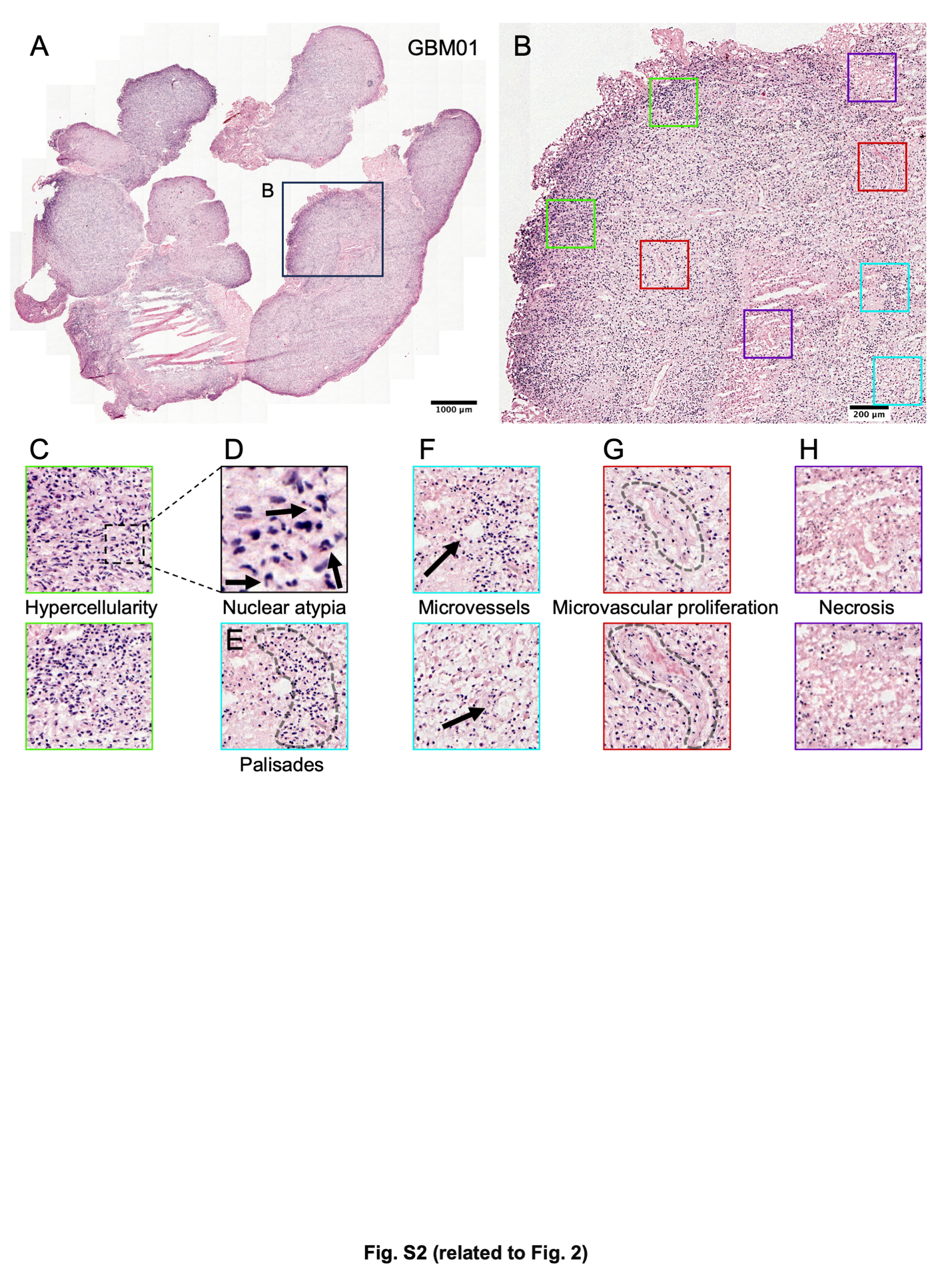


**Figure S2 (data related to Figure 2). Histopathological view in glioblastoma: diffuse, necrotic, microvascular proliferation, microvessel, palisades. (A)** Representative H&E image from GBM01 whole tissue. Rectangle indicates the area represented in B. Bar is 1mm. **(B)** Representative image containing several glioblastoma histopathological hallmarks, highlighted by rectangles color-matched with (C-G). Bar is 200 µm. **(C-G)** Hypercellularity, nuclear atypia, palisades, MV, MVP, necrosis found in (B). Dashed areas indicate palisades and MVP borders in (E, G), respectively. Arrows indicate nuclear atypia of glioblastoma cells (i.e. irregular nuclear shapes that result in chromosome gain/loss, multi-nucleated cells, multi-lobed nuclei) in (D) and MV in (F).


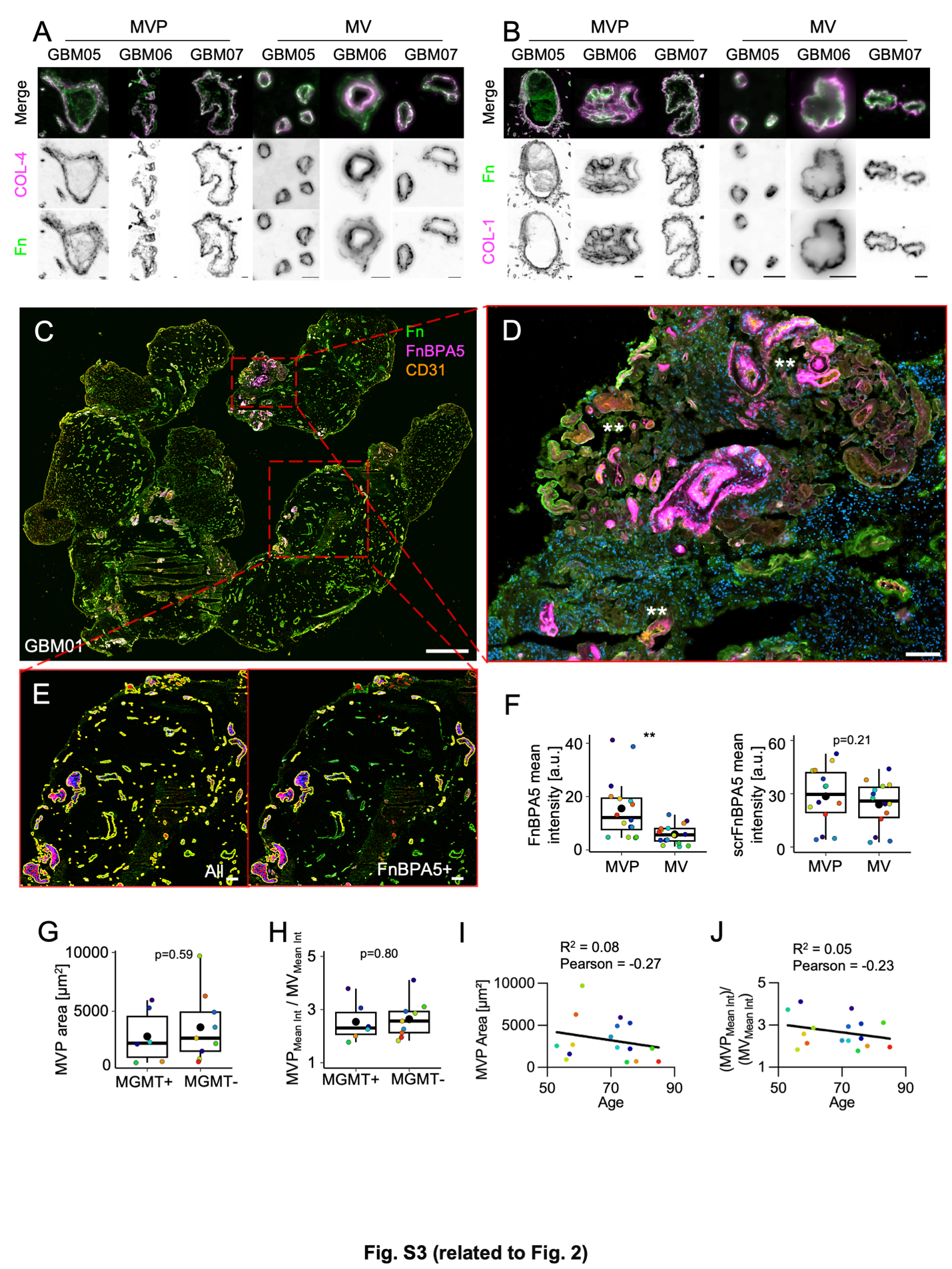


**Figure S3 (data related to Figure 2). (A,B)** Representative MVP and MV crops from whole tissue scans of samples stained for collagen I or collagen IV (magenta) and Fibronectin (green) obtained from 3 selected glioblastomas. Bars are 20 µm. **(C)** Representative image from GBM01 whole tissue scan showing immunostaining for fibronectin (Fn, green), CD31 (orange), and untensed fibronectin fibers as stained by the tension probe FnBPA5 (magenta). Rectangles indicate the areas represented in D, E. Bar is 1mm. **(D)** Representative images indicating vessel structures enriched in untensed fibronectin. Asterisks indicate microvascular proliferations. Bar is 200 µm. **(E)** Analysis utilized for the classification of FnBPA5+ vessels. MV/MVP were detected from whole tissue slide and their morphology (area and circularity) analyzed, then classified FNBPA5+ based on threshold. Left highlights the contours of all the vessels, right the FnBPA5+. Bar is 200 µm. **(F)** FnBPA5 (left, n=16 patients) and scrFnBPA5 (right, n=14 patients) mean values of MVP versus MV. One-tailed unpaired t-tests. **(G)** MVP area of MGMT+ versus MGMT- patients. **(H)** The ratio between FnBPA5 mean intensity in MVP and FnBPA5 mean intensity in MV of MGMT+ versus MGMT- patients indicates no difference in the presence of relaxed fibronectin fibers. **(I)** Low Pearson coefficient between age and MVP area highlights no correlation. **(J)** Low Pearson coefficient highlights no correlation between age and the ratio between FnBPA5 mean intensity in MVP and FnBPA5 mean intensity in MV.


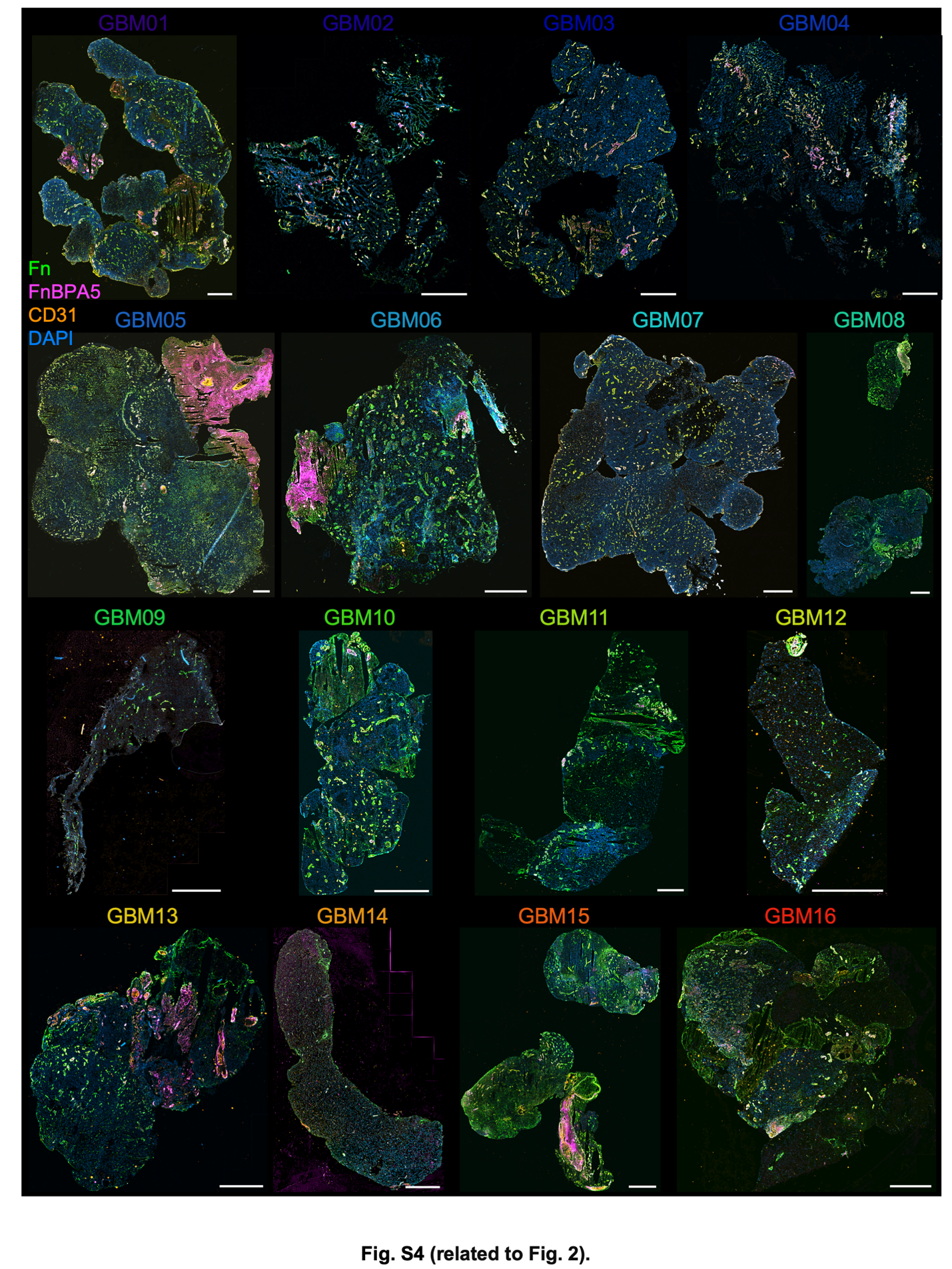


**Figure S4 (data related to Figure 2): Whole tissue scans of the 16 GBM tissue samples used in this study, representing infiltrative tumors in the brain parenchyma.** DAPI (blue), fibronectin (green), CD31 (orange), FnBPA5 (magenta). Bars are 1 mm.


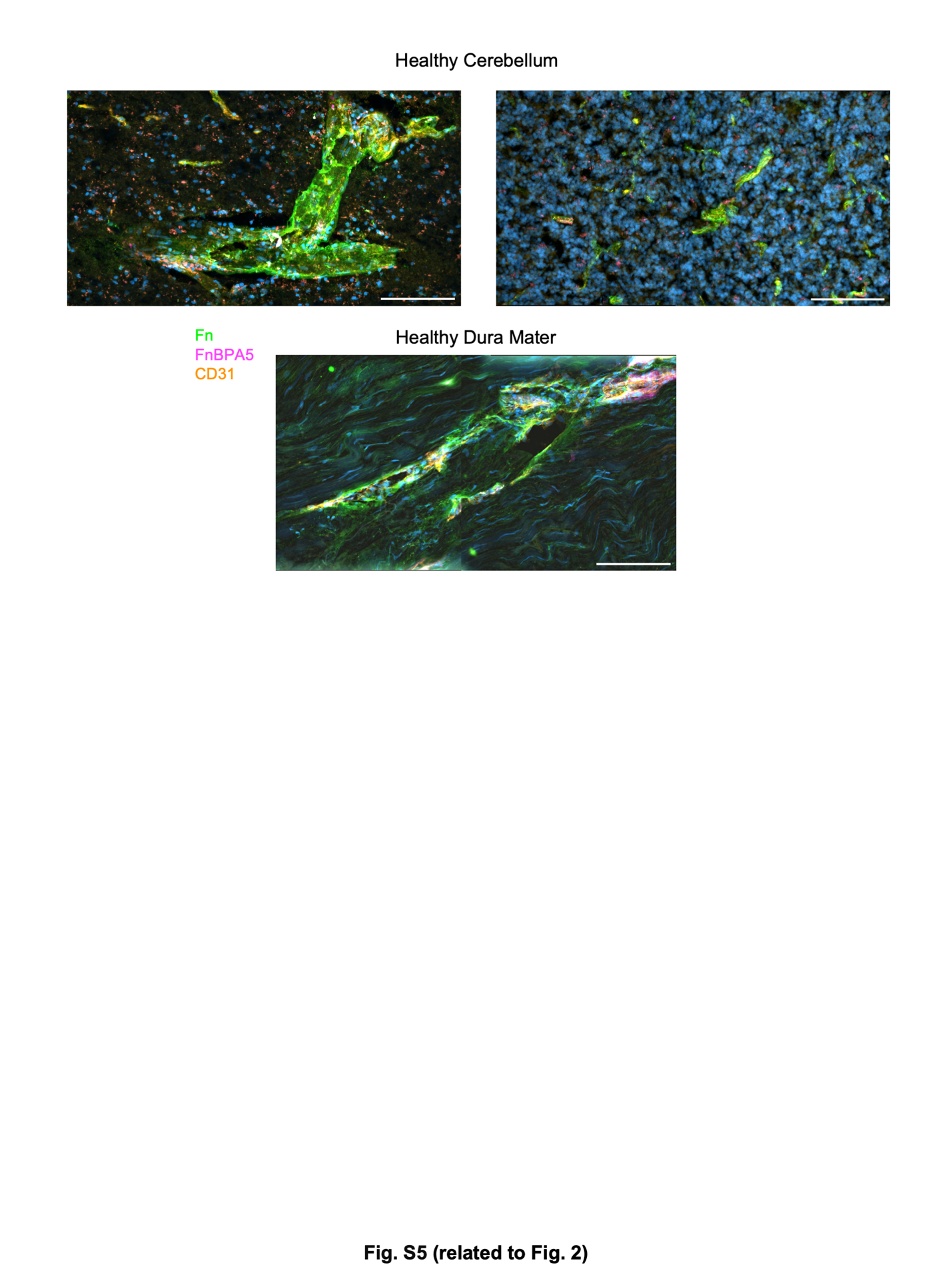


**Figure S5 (data related to Figure 2): Confocal images of Cerebellum and Dura Mater vessels from healthy donors showing no relaxation of fibronectin fibers.** DAPI (blue), fibronectin (green), CD31 (orange), FnBPA5 (magenta). Bars are 100 µm.


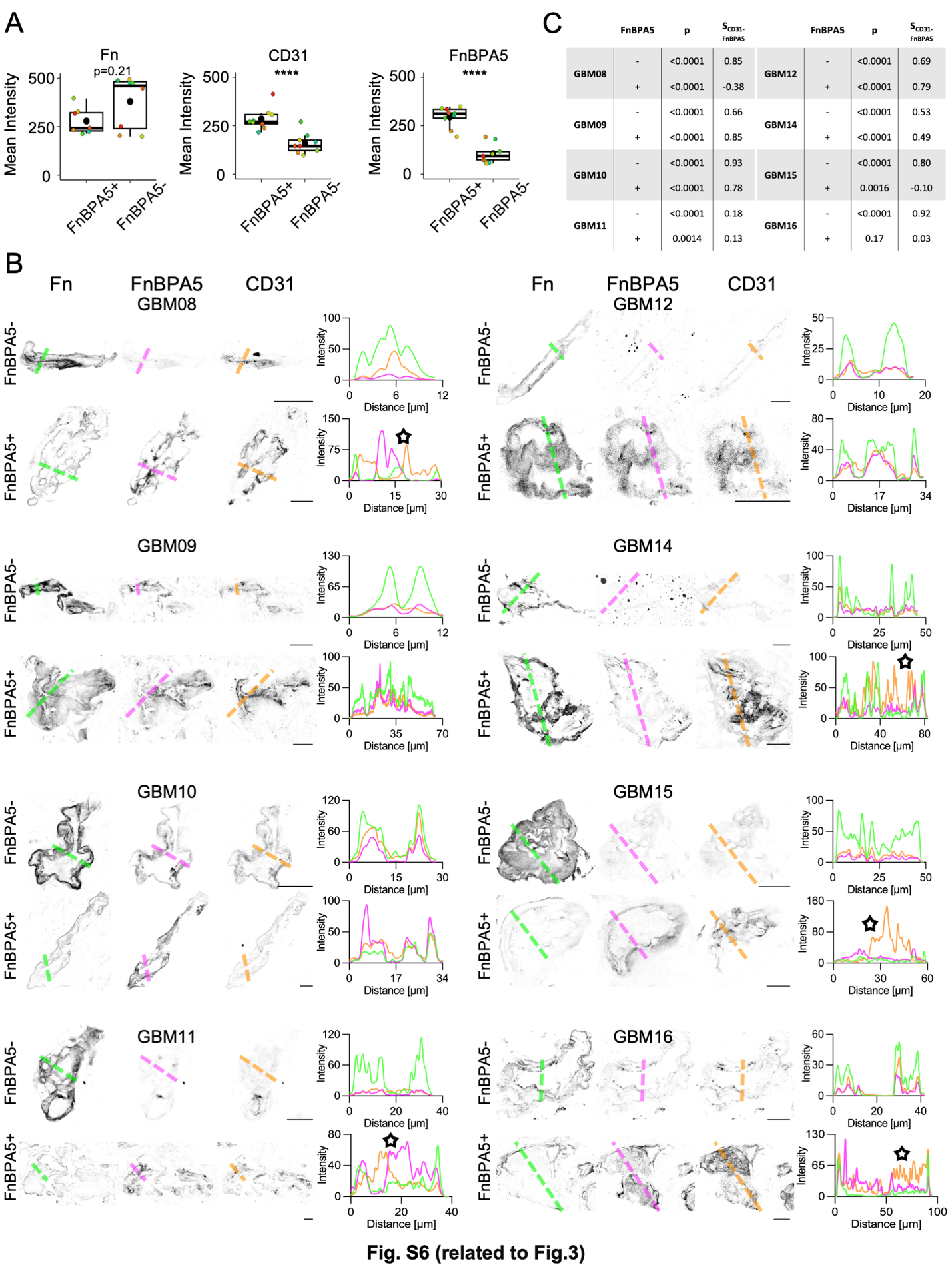


**Figure S6 (data related to Figure 3). (A)** Mean intensity of fibronectin, CD31, FNBPA5 channels in FnBPA5+ MVP versus FnBPA5- MV. **(B)** Spearman correlation values (S_CD31-FnBPA5_) of the CD31 and FnBPA5 line scan trends sampled and visualized in (C) from 8 analyzed tumors. P is the p-value. **(C)** Representative single-channel images of fibronectin, FnBPA5, CD31 in FnBpa5- MV and FnBPA5+ MVP from 8 analyzed tumors (single confocal slices). Lines indicate the sampled areas for the intensity profiles and are color-matched with the respective channels - fibronectin (green), CD31 (orange), and FnBPA5 (magenta). Juxtaposition between CD31 and FnBPA5 in the graphs is highlighted with a black star. Bars are 20 µm.


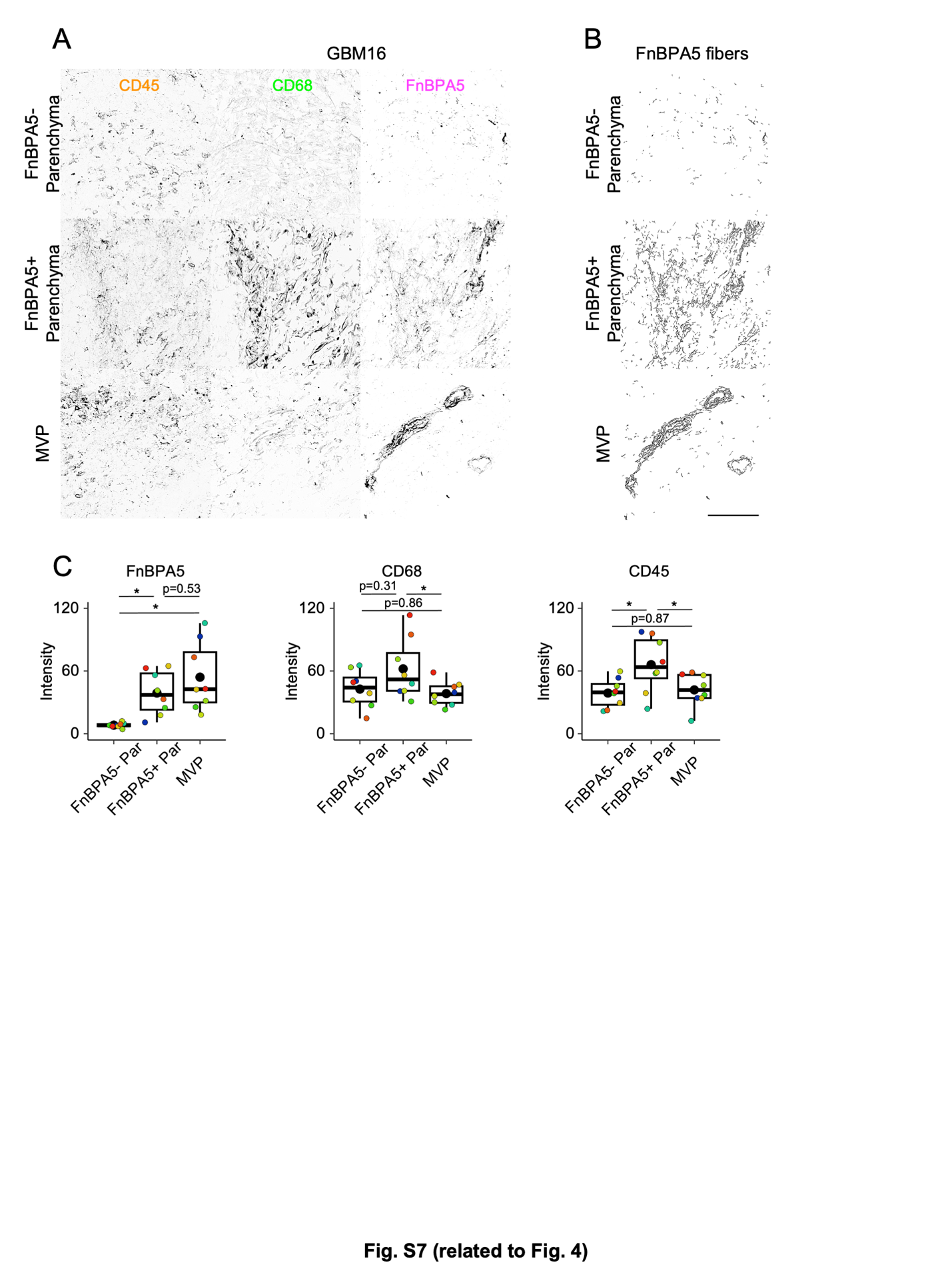


**Figure S7 (data related to Figure 4).** **(A)** Representative confocal images of CD45 (orange, left), CD68 (green, middle), FnBPA5 (magenta, right) channels in the tumor GBM16 when the parenchymal ECM is devoid of untensed fibronectin fibers (top), rich in disorganized, untensed fibronectin fibers (FnBPA5+, middle), rich in untensed fibronectin fibers organized in a vessel-like fashion (MVP, bottom). **(B)** Representative binarized rendering of the segmented untensed fibronectin fibers in FnBPA5- parenchyma, FnBPA5+ parenchyma, MVP. **(C)** Mean intensity of FnBPA5 (left), CD68 (middle), CD45 (right) channels in FnBPA5- parenchyma, FnBPA5+ parenchyma, MVP. RM one-way ANOVA test with Geisser-Greenhouse correction and Turkey’s multiple comparison test. Non-statistically significant p-values reported as 2-digit numbers.


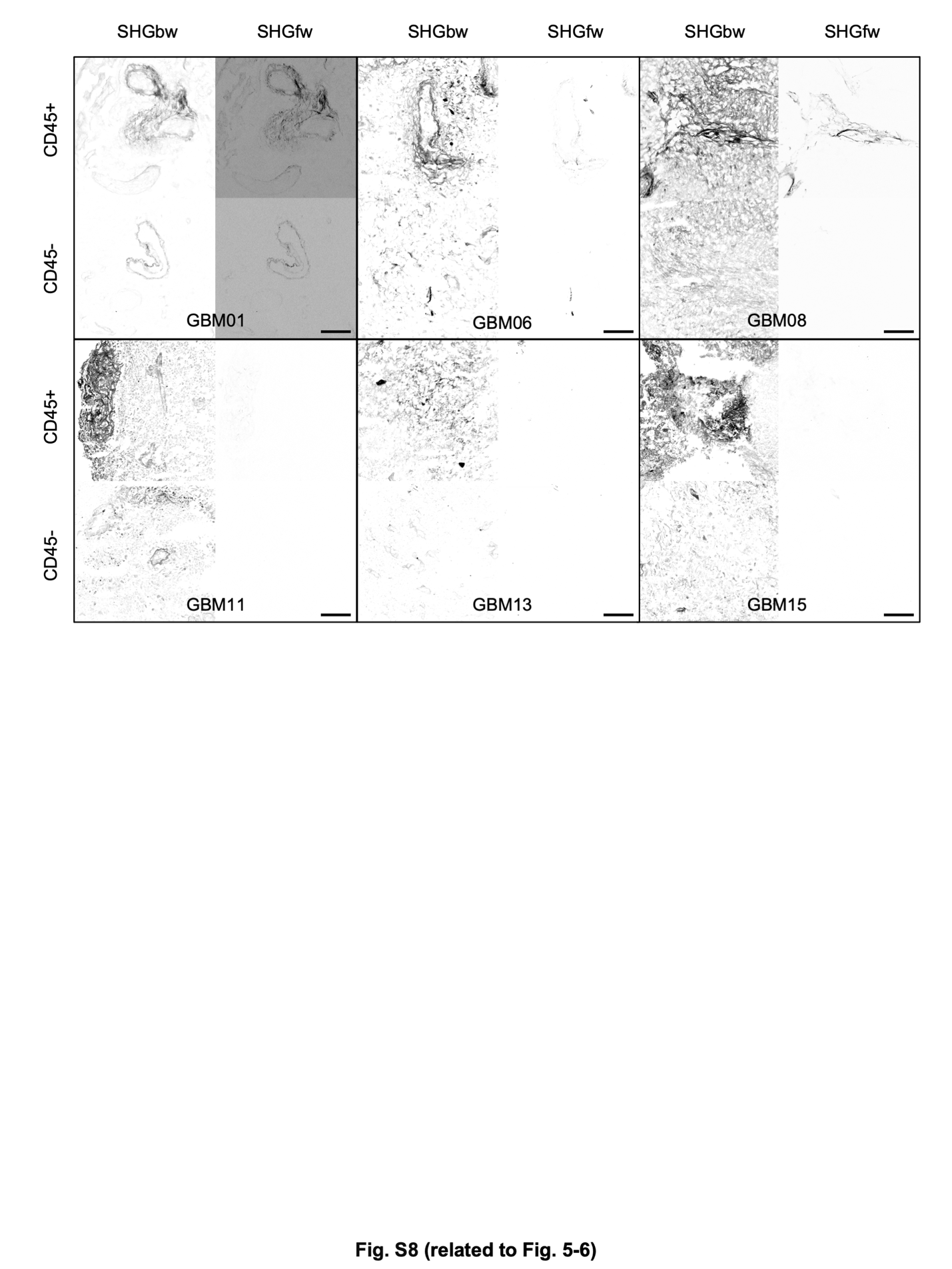


**Figure S8 (data related to Figure 5-6).** Representative confocal images of SHGbw (backward scattered, left in each panel) and SHGfw (forward scattered, right in each panel) of the 6 tumors displaying CD45+ cell aggregates. CD45+: areas with the representative aggregate (red squares in Figure 5); CD45-: representative area devoid of CD45 (dotted red squares in Figure 5). Bars are 100 µm.


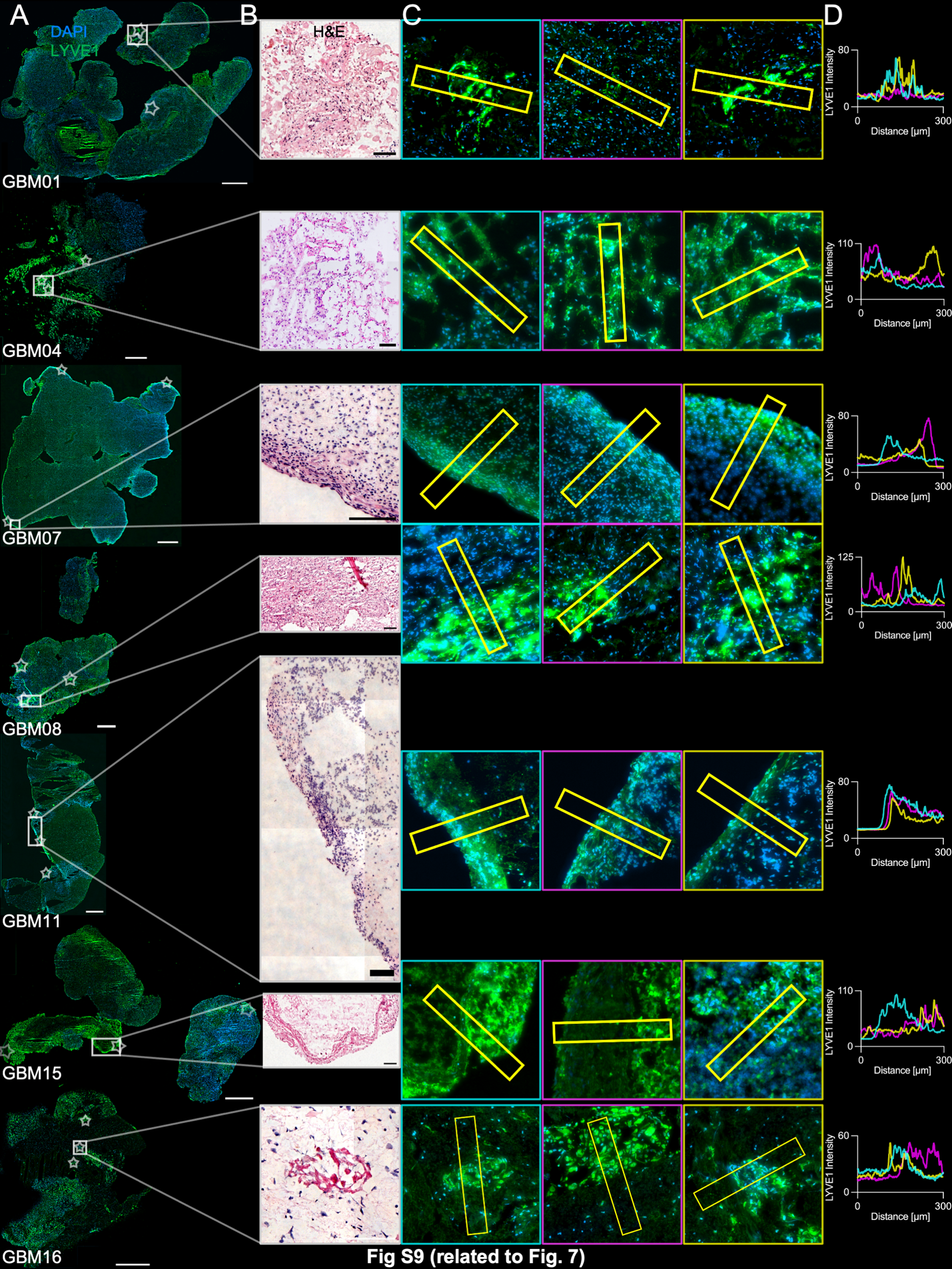


**Figure S9 (data related to Figure 7). (A)** Whole tissue scan of the 7 glioblastoma tissue samples stained for DAPI (blue), LYVE1 (green) and containing LEC-like cells (the 7 tissues non-containing LEC-like cells are not shown). Stars indicate the FOVs sampled 50x300 µm areas shown in (C). Bars are 1 mm. **(B)** H&E stainings of the panels reported in Figure 7E, F. Bars are 100 µm. **(C)** FOVs sampled with 50x300 µm areas (yellow rectangles) corresponding to the line profiles in (D). Panel borders are color matched with the trends in (D). Bars are 100 µm. **(D)** Line scan profiles from the areas in (C).


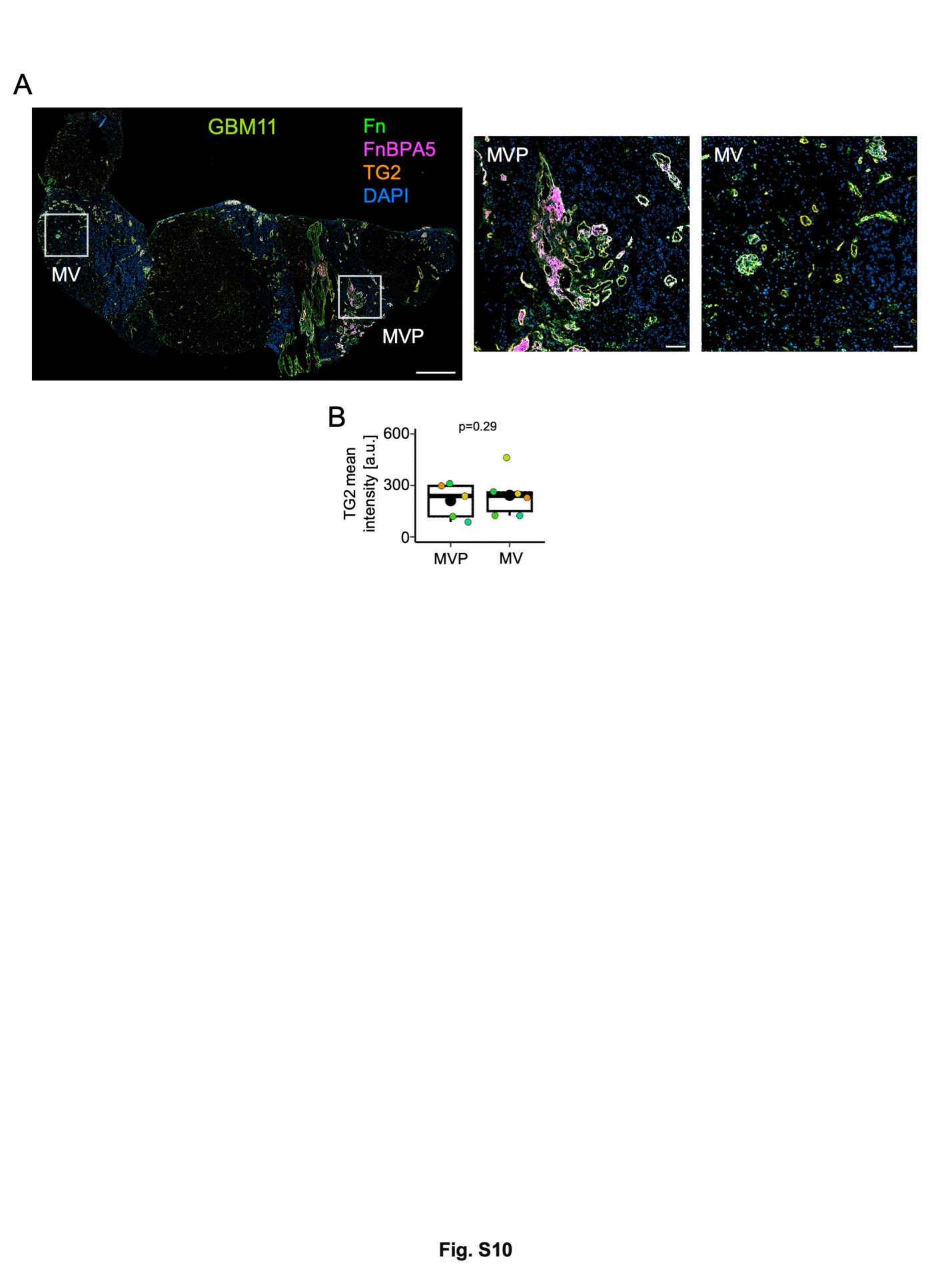


**Figure S10: (A)** Whole tissue scan of sample coming from patient GBM11 stained for fibronectin (green), FnBPA5 (magenta), TG2 (orange) and DAPI (blue). Scale bar: 1mm. Zoom in image showing MVP and MV regions. Scale bar: 100 µm. **(B)** Quantification of TG2 content in regions highly enriched in untensed fibronectin fibers (MVP) versus regions lacking untensed fibronectin fibers (MV). Two-tailed unpaired t-test, non-statistically significant p-value is reported. 1 dot represents the average from 1 patient sample and the bigger, black dots represent the mean value.
